# Supplementary material for: Mapping the interaction surface between CaVβ and actin and its role in calcium channel clearance
Source: Nat Commun. 2025 May 10;16:4352. doi: 10.1038/s41467-025-59548-x (PMC12065904; doi:10.1038/s41467-025-59548-x)
Supplement: Supplementary file 1 — Supplementary Information [file 41467_2025_59548_MOESM1_ESM.pdf]

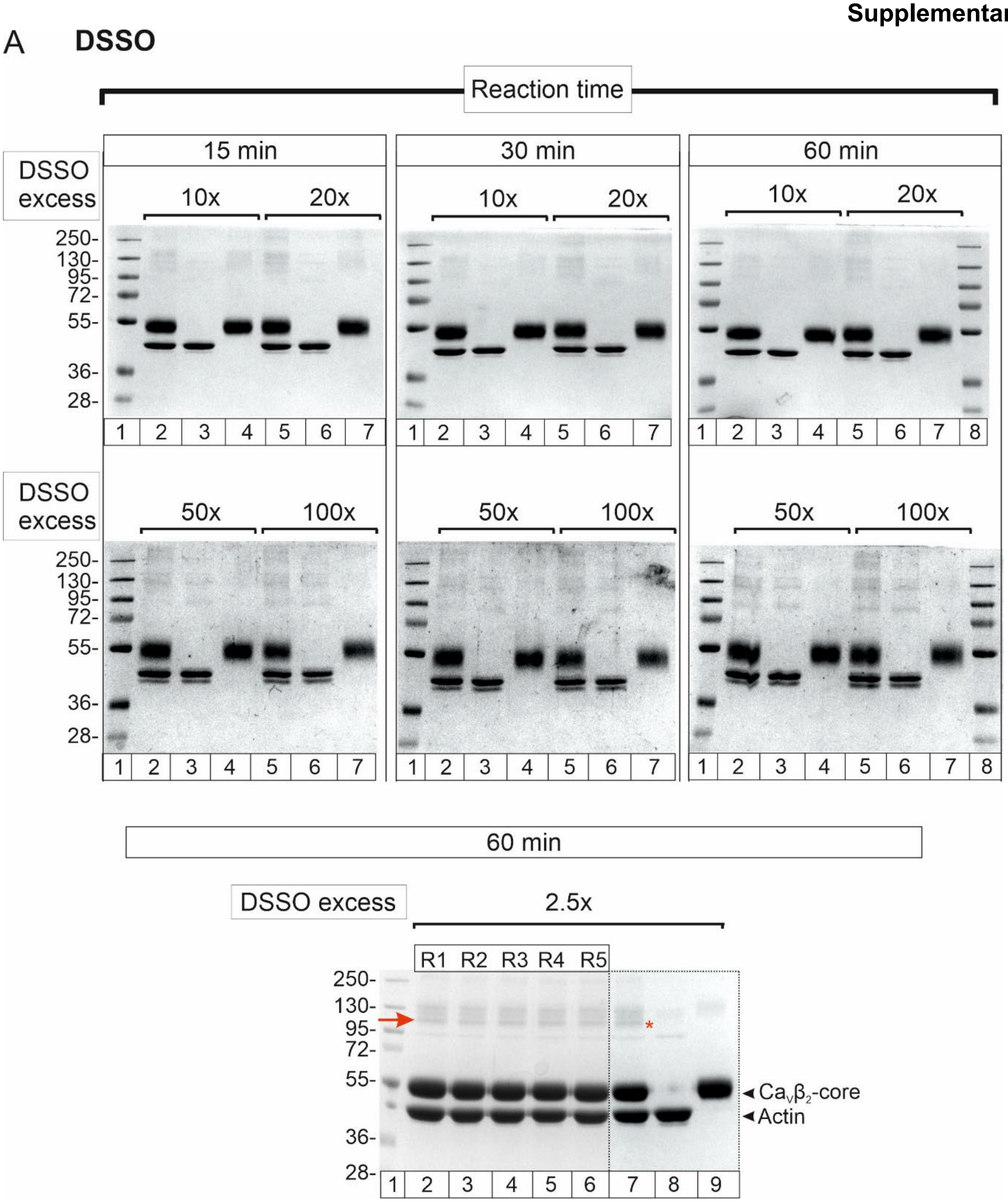

Supplementary Figure 1. Summary of the optimization for the cross-linking reaction conditions of $Ca_V\beta$ and actin with DSSO and DSBU cross-linkers. A, Top panel, images of the SDS-PAGE gels for cross-linking reactions between $Ca_V\beta_2$-core and actin with DSSO at molar excess of ≥ 10 (10x, 20x, 50x, 100x) and different reaction times (15, 30 and 60 min) at RT. For each gel (1.0 mm thickness); lane 1, molecular weight standards with numbers denoting the molecular masses in kDa; lanes 2 and 5, $Ca_V\beta$–actin cross-linking reaction; lanes 3 and 6, actin alone with cross-linker and lane 4 and 7, $Ca_V\beta$ alone with cross-linker. Bottom panel, SDS-PAGE gel (1.5 mm thickness) of the optimal DSSO cross-linking reaction for $Ca_V\beta_2$-core–actin. A 60-minute reaction time at lower molar excess (2.5x) was found to be the optimal condition for DSSO. The red arrow and asterisk denote the gel band excised and subjected to LC-MS/MS analysis. This band is not visible in control reactions where either each protein was incubated alone with the cross-linker or no cross-linker was added. The replicates analyzed R1 to R5 (lanes 2-6) are documented in Supplementary Table 2. The dotted line box (lanes 7-9) encloses the gel image shown in Figure 2C, main text.

Supplementary Figure 1B

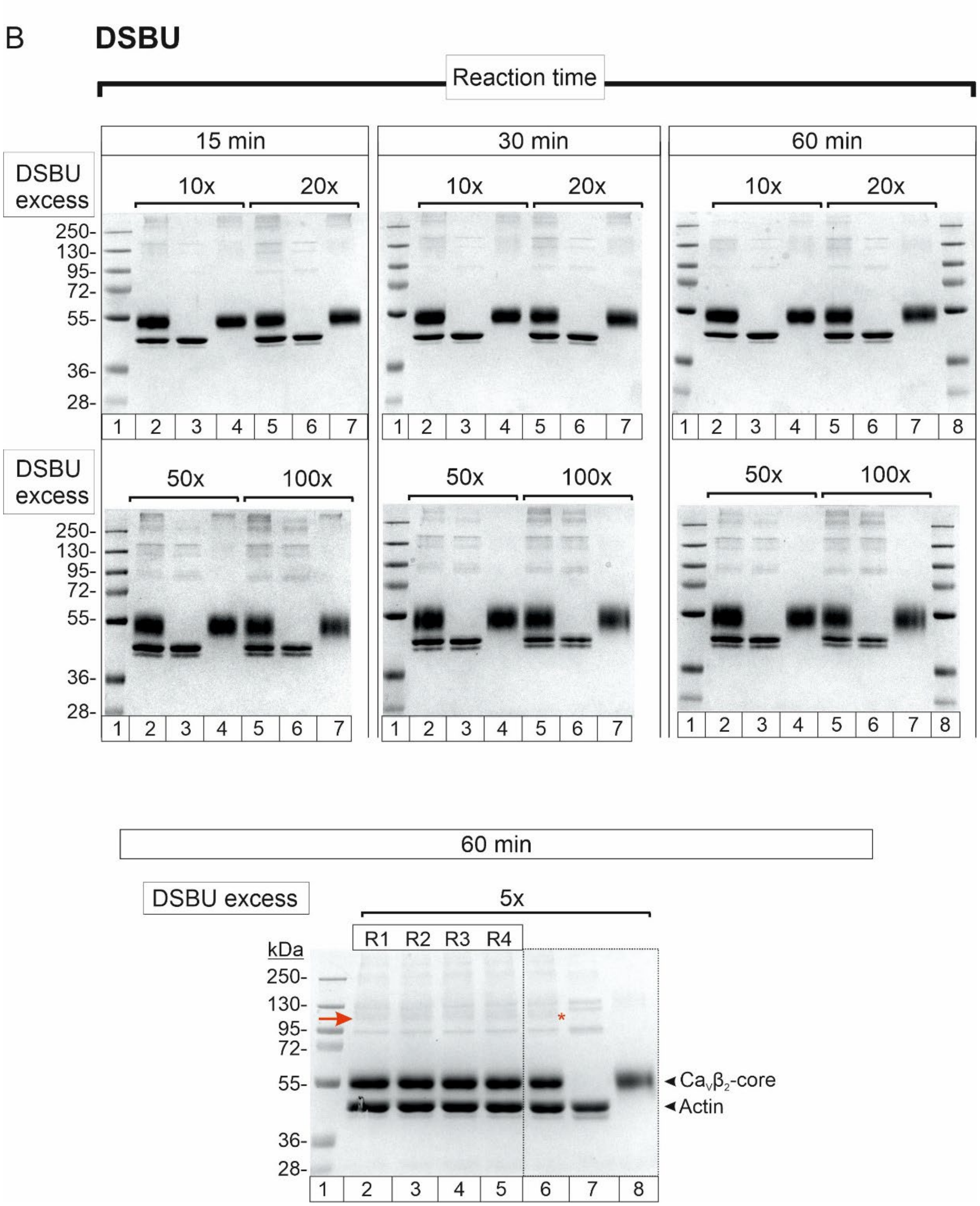

Supplementary Figure 1. Summary of the optimization for the cross-linking reaction conditions of $Ca_V\beta$ and actin with DSSO and DSBU cross-linkers. B, Top panel, same as *A*, but for DSBU. Bottom panel, SDS-PAGE gel (1.5 mm thickness) of the optimal DSBU cross-linking reaction for $Ca_V\beta_2$-core–actin that occurred at a molar excess of 5.0x during a 60-minute reaction time. The replicates analyzed R1 to R4 (lanes 2-5) are documented in Supplementary Table 2. The dotted line box (lanes 6-8) encloses the gel image shown in Figure 2C, main text.

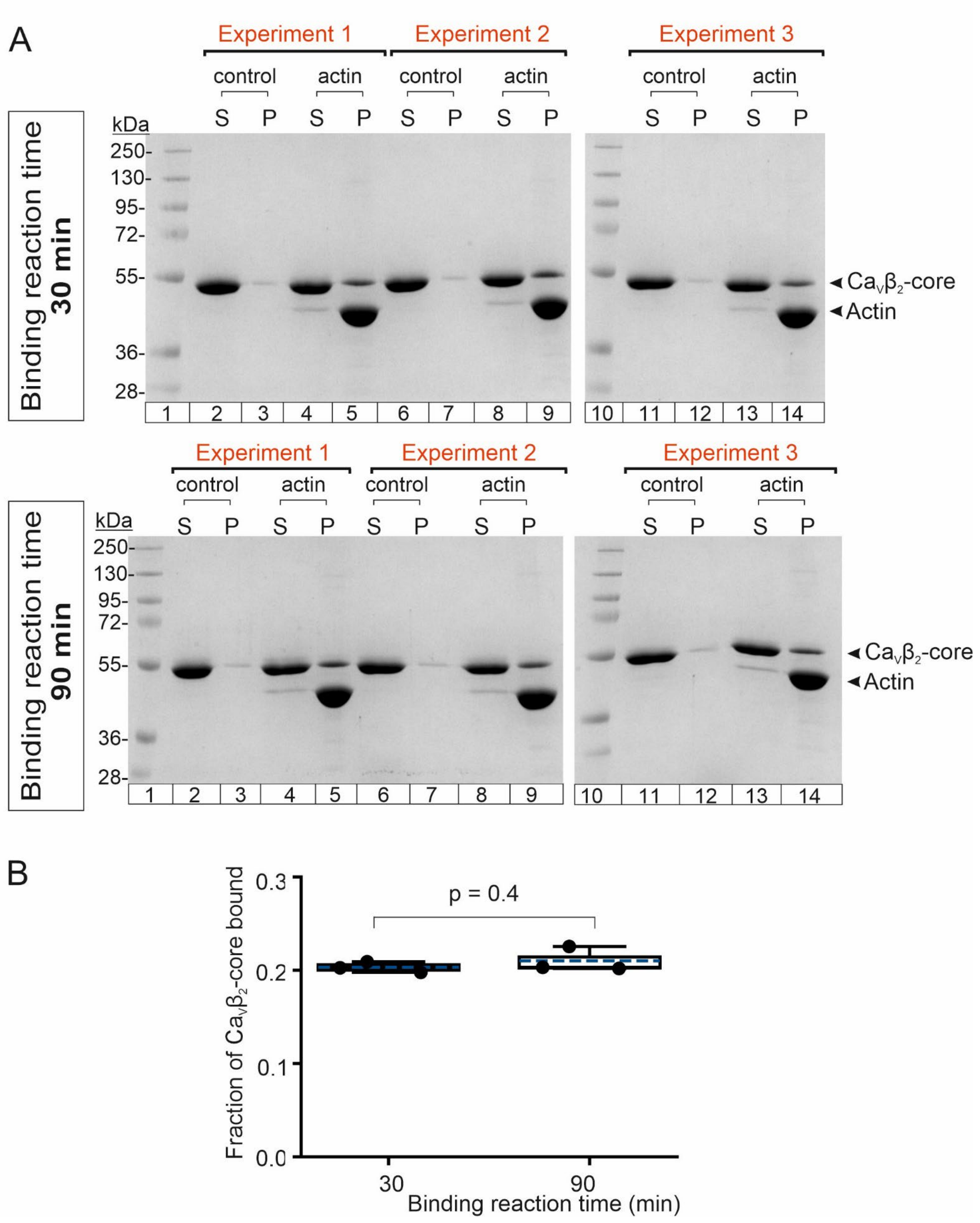

Supplementary Figure 2. Stability of $Ca_V\beta_2$–actin complex formation over time assessed by F-actin cosedimentation assay. **A**, Images of the SDS-PAGE for F-actin cosedimentation assays with $Ca_V\beta_2$-core for binding reaction times of 30 minutes (upper panel) and 90 minutes (lower panel). For each gel, lane 1, molecular weight standards in kDa; S, supernatant; P, pellet. Control and actin denote that the assay was performed in the absence and presence of actin, respectively. Each assay was repeated three times (experiments 1 to 3). **B**, Box plot of the fraction of the $Ca_V\beta_2$-core bound to actin after 30 and 90 minute reaction showing the mean (dashed line), median (continuous line), interquartile range (25th–75th percentiles, box edges) and whiskers (1.5× interquartile range). Each dot represents a separate experiment (n = 3). p-value was determined by two-sided t-test.

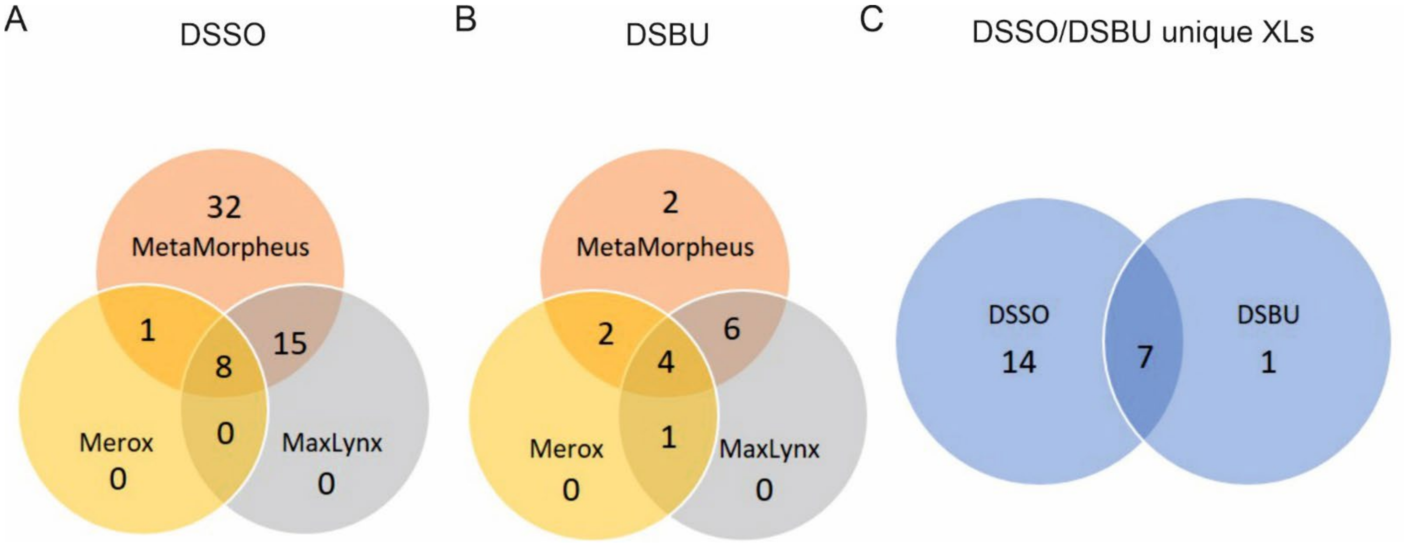

Supplementary Figure 3. Venn diagrams of the number of intermolecular crosslinks (inter-XLs) between $Ca_V\beta_2$ and actin. **A**, Venn diagram of the total intermolecular XLs identified by the three software packages used (MetaMorpheus, MaxLynx and Merox) in five replicates with DSSO as crosslinker. **B**, same as *A*, but for the four replicates with DSBU. **C**, Venn diagram indicating the overlap between unique intermolecular XLs obtained with DSSO and DSBU. Data taken from Supplementary Table 2.

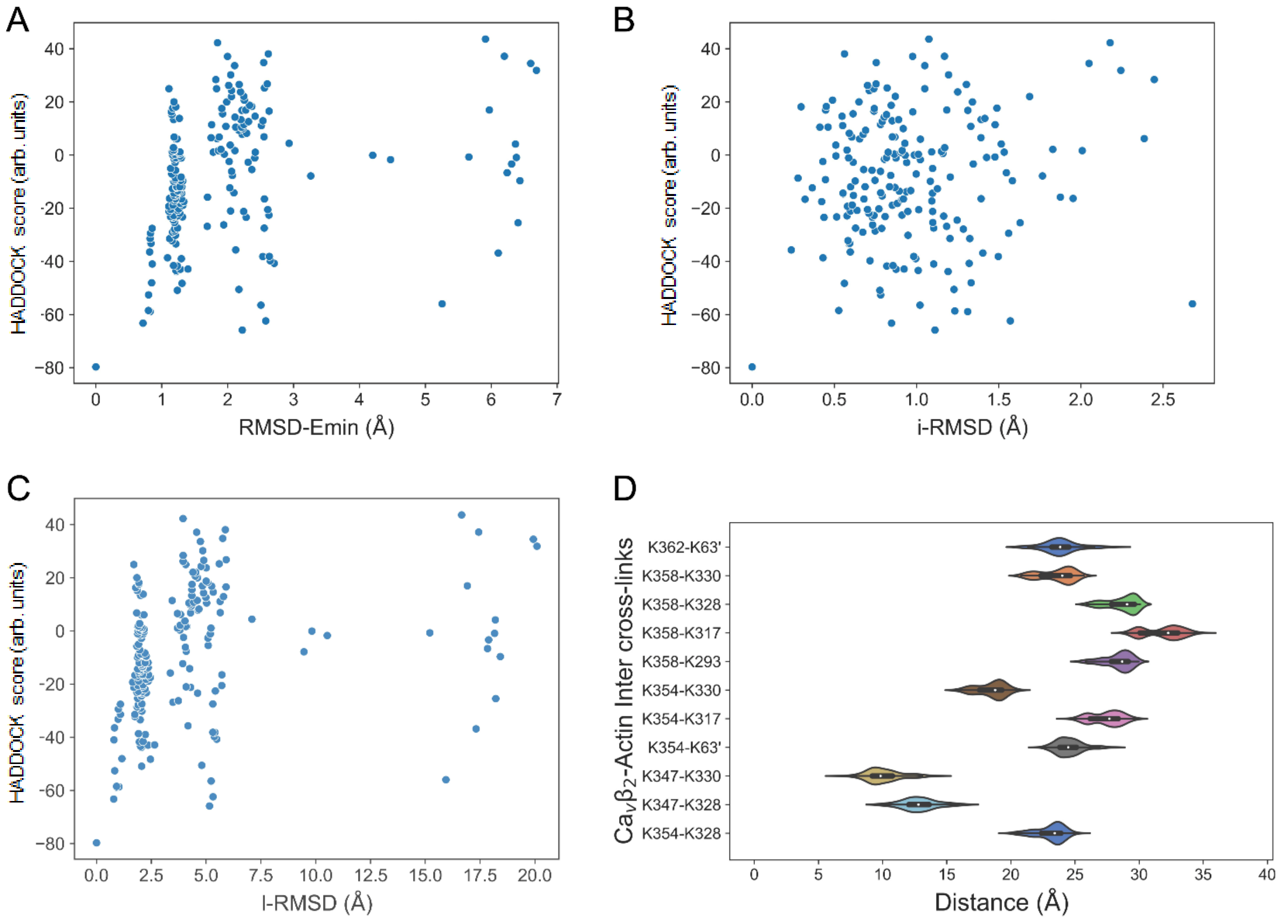

Supplementary Figure 4. Model docking data for $Ca_V\beta_2$ and actin. **A**, HADDOCK score versus global RMSD (RMSD-Emin) plot for the sole cluster obtained for the $Ca_v\beta_2$–actin docking containing 198 out of 200 generated models. The global RMSD is calculated including the alpha carbon atoms of all residues of both proteins, using as reference the structure with the lowest HADDOCK score. **B**, HADDOCK score versus interface RMSD (i-RMSD) plot for $Ca_v\beta_2$–actin docking structures. The interface RMSD is calculated including the backbone atoms of all interface residues of both proteins, after superimposing these interfacial residues, which are defined using an interatomic distance of 10 Å. The reference structure is the one with the lowest HADDOCK score. **C**, HADDOCK score versus ligand RMSD (l-RMSD) plot for $Ca_v\beta_2$–actin docking structures. The ligand RMSD is calculated including the backbone atoms of $Ca_V\beta_2$ (as ligand) after fitting on the actin dimer and using as reference the structure with the lowest HADDOCK score. **D**, Mapped Euclidean Cα-Cα cross-linked pair distance distribution for the 198 docking structures belonging to the best cluster generated by HADDOCK 2.4 for $Ca_v\beta_2$–actin.

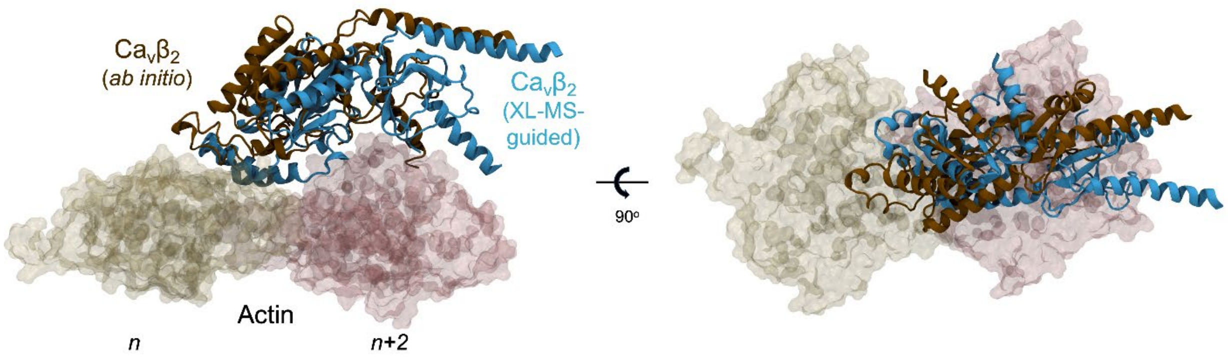

Supplementary Figure 5. *Ab initio* docking model with the lowest I-RMSD compared to the best scored XL-MS-guided model of the $Ca_V\beta_2$-actin complex. Superimposition of backbone heavy atoms of actin-bound $Ca_V\beta_2$ structures generated with the two different docking approaches (brown and cyan for the *ab initio* and XL-MS-guided models, respectively). The two actin subunits used as reference for the alignment are shown in space-filling mode (yellow and pink, respectively). Based on the HADDOCK score, the *ab initio* docking model was ranked as number 208 out of 400 models in the respective control simulation (see Supplementary Table 6). The I-RMSD between the two actin-bound $Ca_V\beta_2$ structures is 14.0 Å, whereas the i-RMSD is 7.9 Å, considering actin and $Ca_V\beta$ residues found to be at the interface (i.e. within 10 Å of the other protein partner) for both models, which comprise 73% of the PPI surface.

Supplementary Figure 6

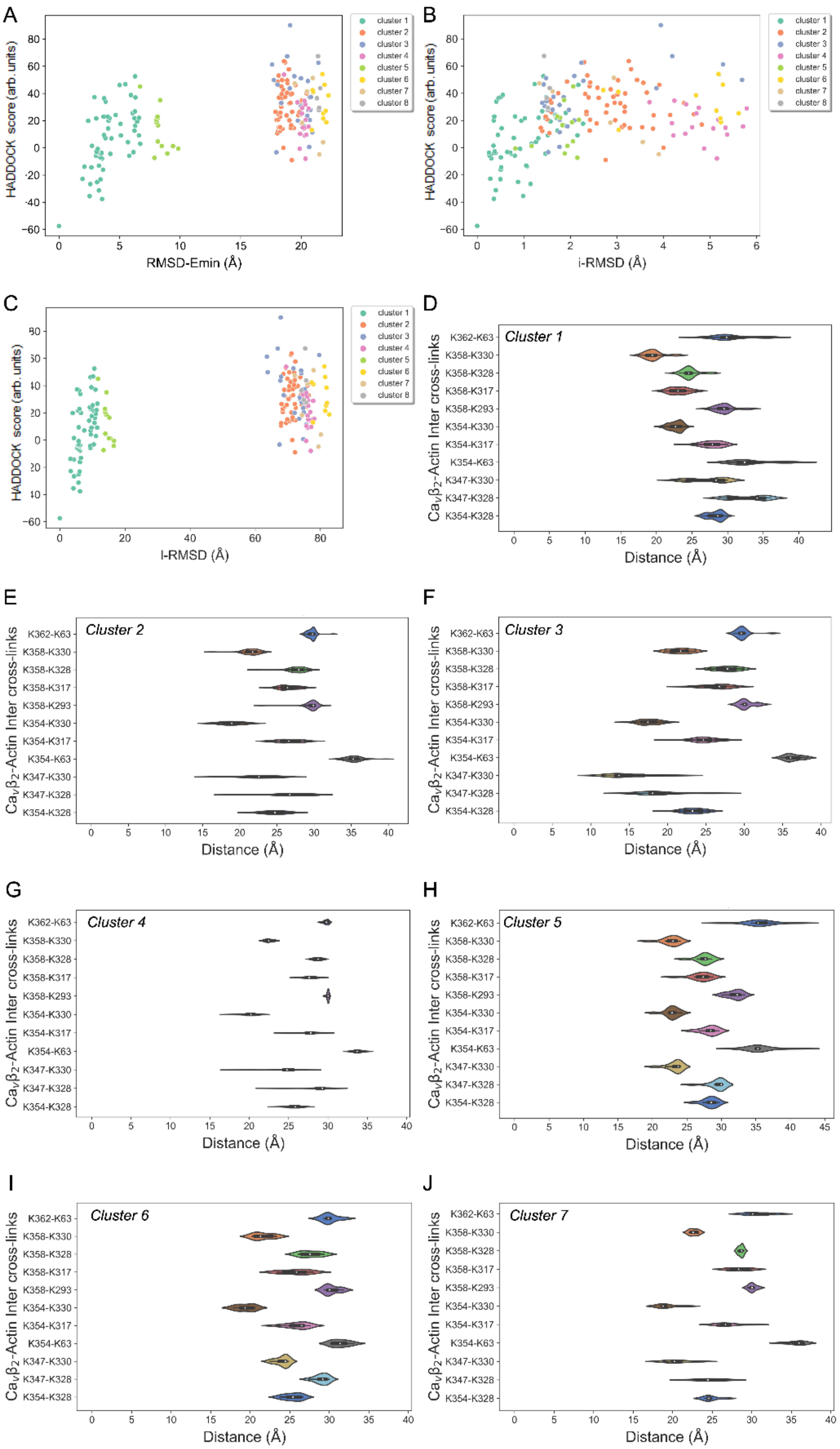

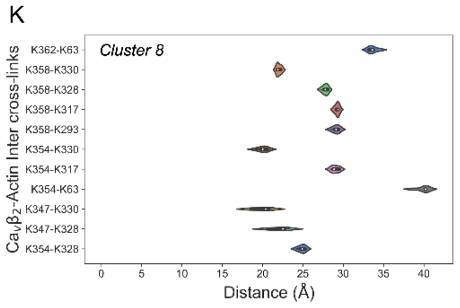

Supplementary Figure 6. Model docking data for Ca$_V$β$_2$-and actin monomer. **A-C**, Plots for the HADDOCK score of Ca$_V$β$_2$-actin monomer docking structures *versus* global RMSD (RMSD-Emin, A), interface RMSD (i-RMSD, B) and ligand RMSD (l-RMSD, C). **D-K**, Mapped Euclidean Cα-Cα cross-linked pair distance distribution for the docking structures for each of the generated clusters. A-C, calculated as explained in Supplementary Figure 4. The reference structure for all RMSD calculations is the one with the lowest HADDOCK score. The cluster size is given in Supplementary Table 4C.

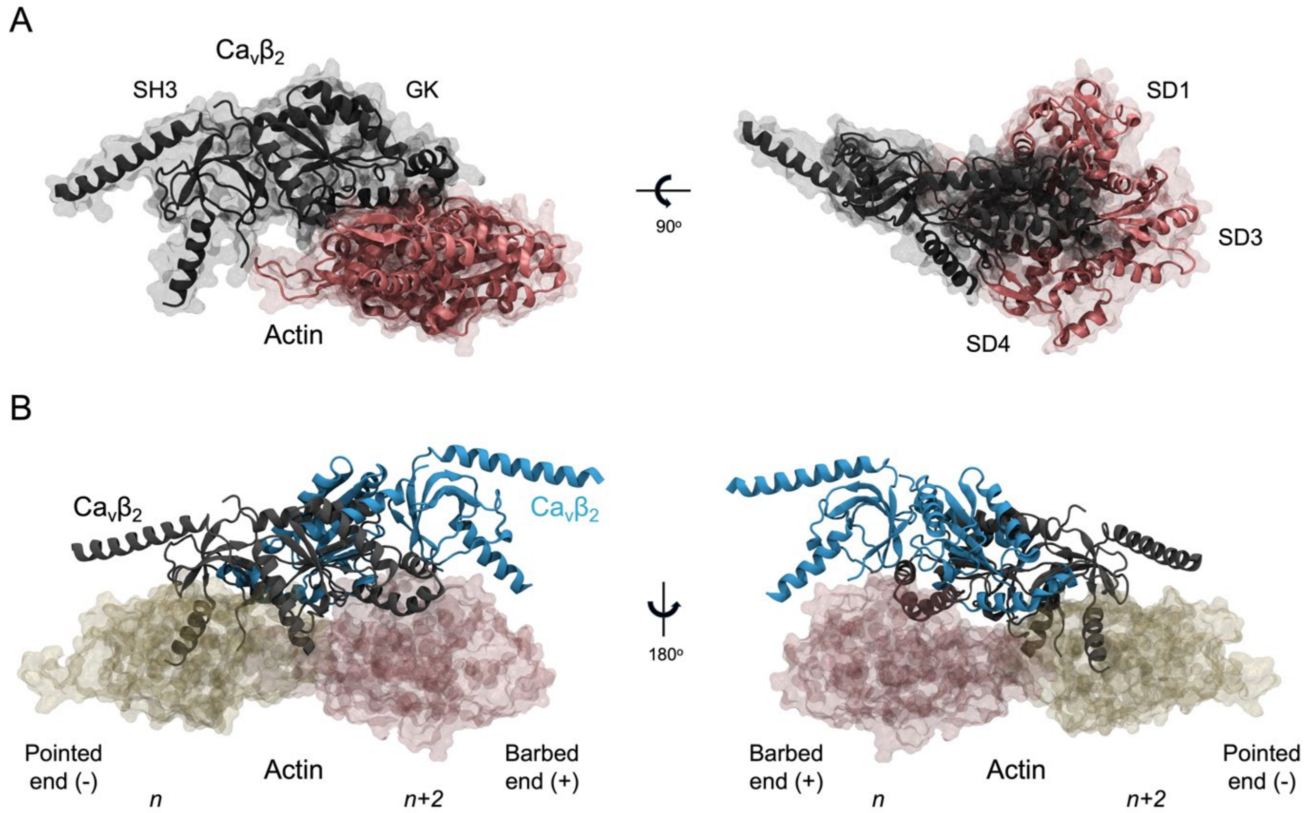

Supplementary Figure 7. Structural docking model of $Ca_V\beta_2$ bound to an actin monomer. A, Space-filling and ribbon diagram showing two views related by a 90° rotation of the best-scoring docking model of $Ca_V\beta_2$ (gray) complexed with an actin monomer (pink). **B**, Superimposition of backbone heavy atoms of actin-bound structures of $Ca_V\beta_2$. Each $Ca_V\beta$ structure represents the average of the top four models of the best-scored cluster generated by docking to actin, either monomeric or dimeric (gray and blue ribbons, respectively). The two actin subunits are shown in space-filling model (yellow and pink); the *n+2* actin subunit was used as reference for the alignment.

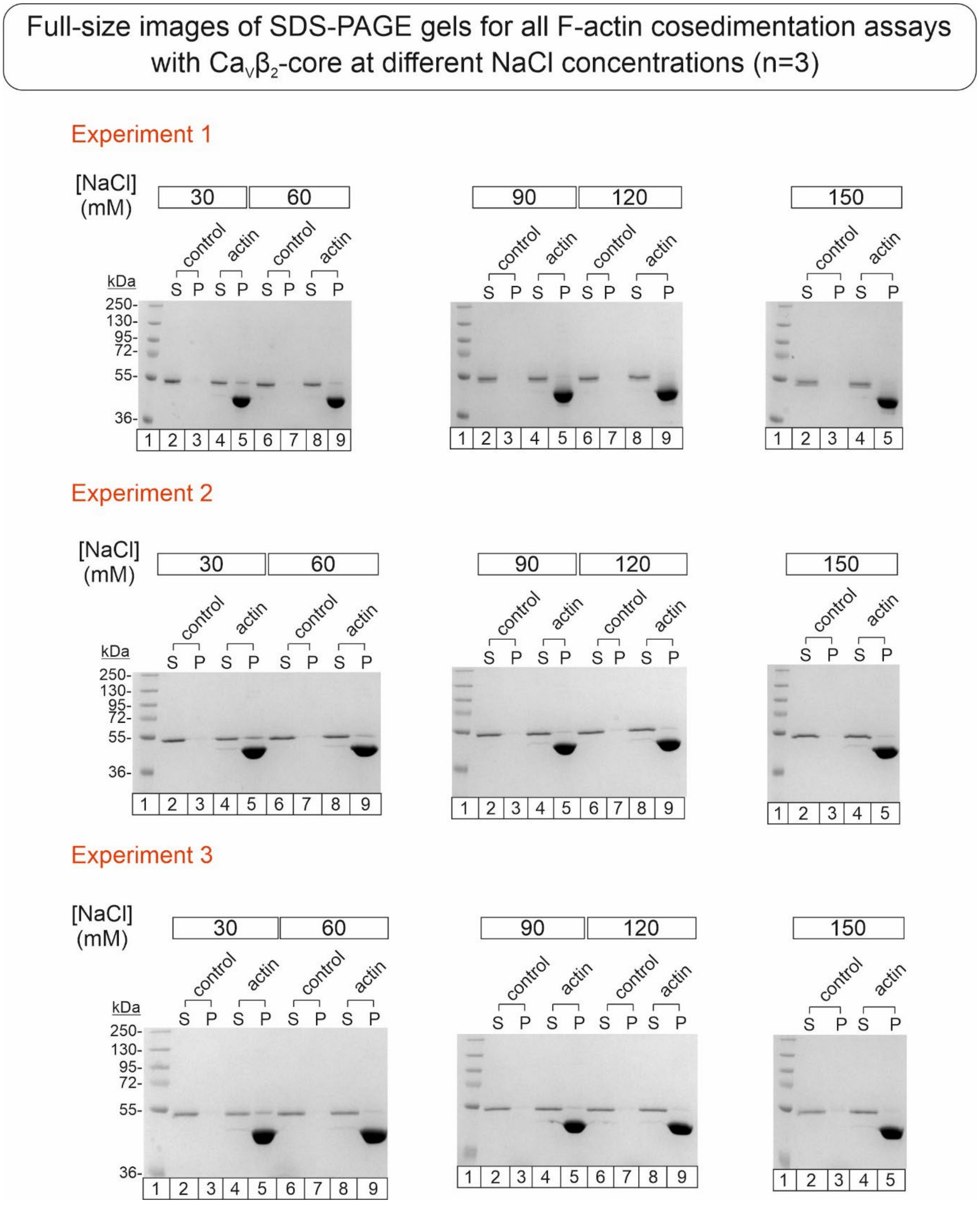

Supplementary Figure 8. Binding of $Ca_V\beta_2$-core to actin at different NaCl concentratios. Full-size images of the SDS-PAGE gels for all F-actin cosedimentation assays with $Ca_V\beta_2$-core at the indicated NaCl concentration (n=3 for each NaCl concentration). For each gel, the NaCl concentration used in the assay is shown in a box; S and P denote supernatant and pellet, respectively; and control and actin denote that the assay was performed in the absence ($Ca_V\beta_2$-core alone) and presence of actin, respectively. The same molecular weight standards were used in each experiment, with the molecular masses in kDa shown adjacent to the left-most gel.

Supplementary Figure 9

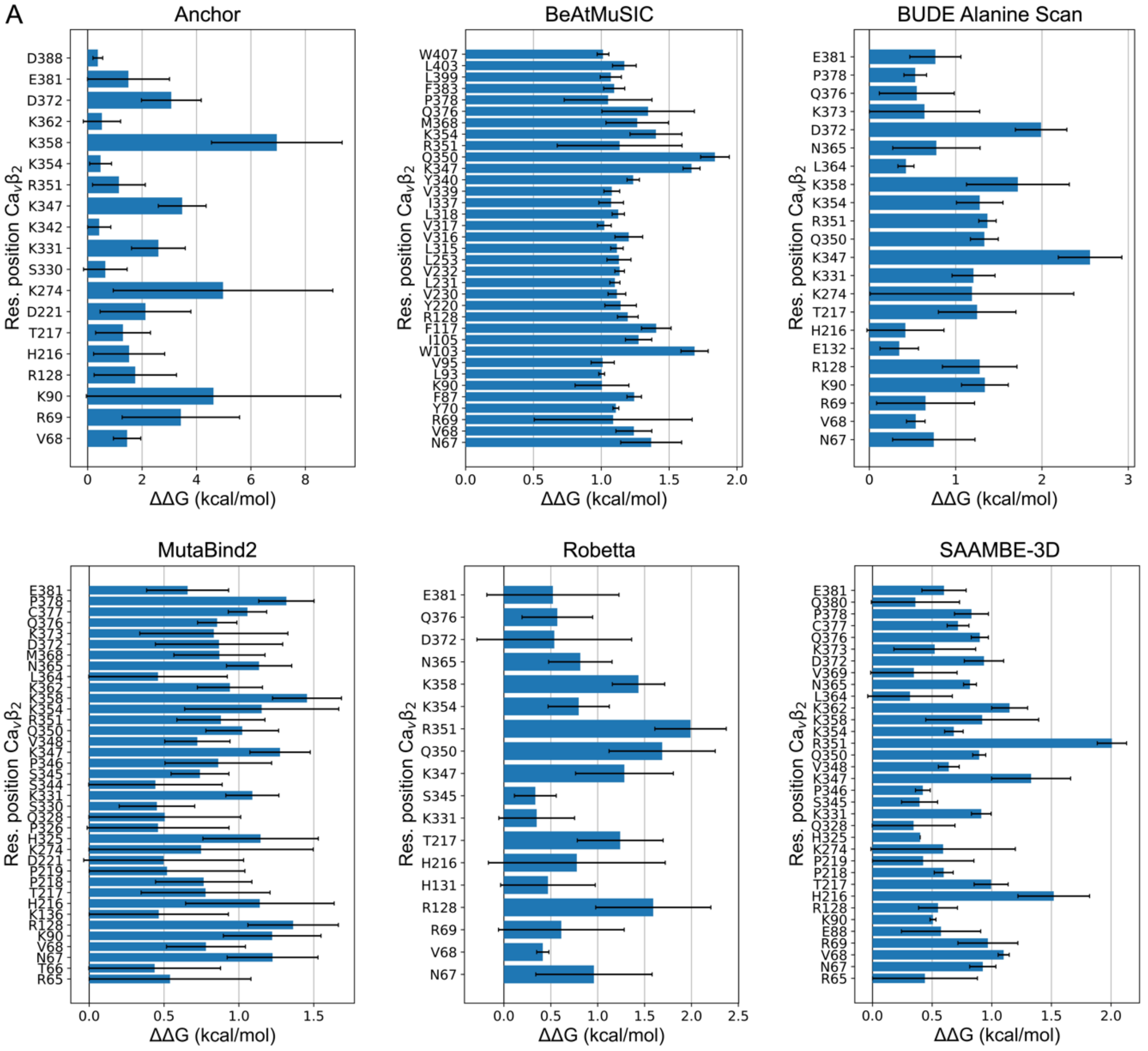

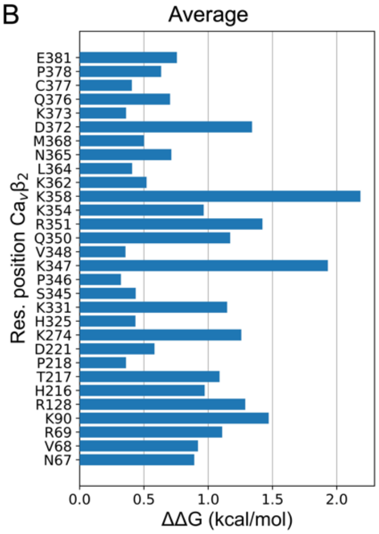

Supplementary Figure 9. Computational alanine scanning analysis to identify hotspot residues on Ca$_V$β$_2$ at its actin interaction interface. A, Computational alanine scanning of actin interface residues on Ca$_V$β$_2$ was performed on a set of six webservers, as indicated: Anchor, BeAtMuSiC, BUDE Alanine Scan, Mutabind2, Robetta and SAAMBE-3D using the top four structures of the best docking cluster. For each predictor, changes in binding free energy upon alanine mutation of the corresponding Ca$_V$β$_2$ residue (ΔΔG) were averaged across the four selected docking models. Error bars correspond to the standard deviation. **B**, ΔΔG values averaged over all six predictors. Only mutations resulting in a ΔΔG higher than 0.3 kcal/mol are shown.

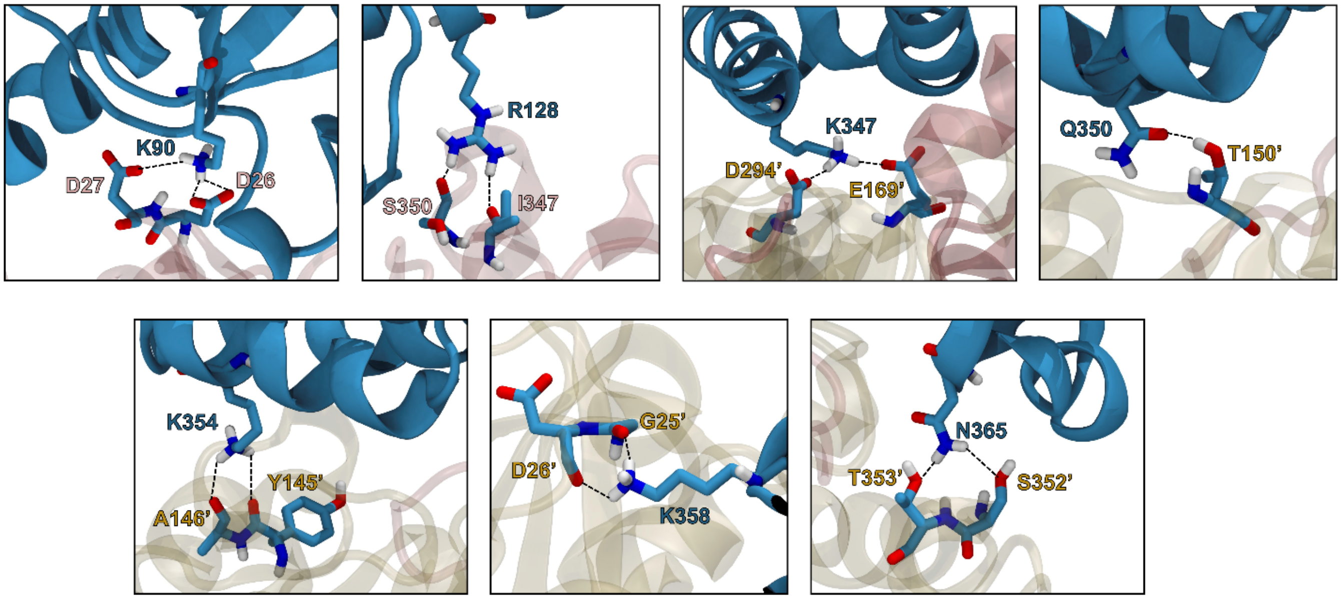

Supplementary Figure 10. Short-range interactions formed by $Ca_V\beta_2$ hotspot residues at the protein-protein interface with actin. The best scored model of the $Ca_V\beta_2$-actin complex was used for the visual inspection of the protein-protein interactions. The two proteins are shown as ribbons, with $Ca_V\beta_2$ and hotspot residues in blue, and the two actin subunits and interacting residues in light pink and grey (*n*+2 and *n* monomers, respectively). Residues are displayed as sticks and interactions are indicated with dashed lines.

Supplementary Figure 11

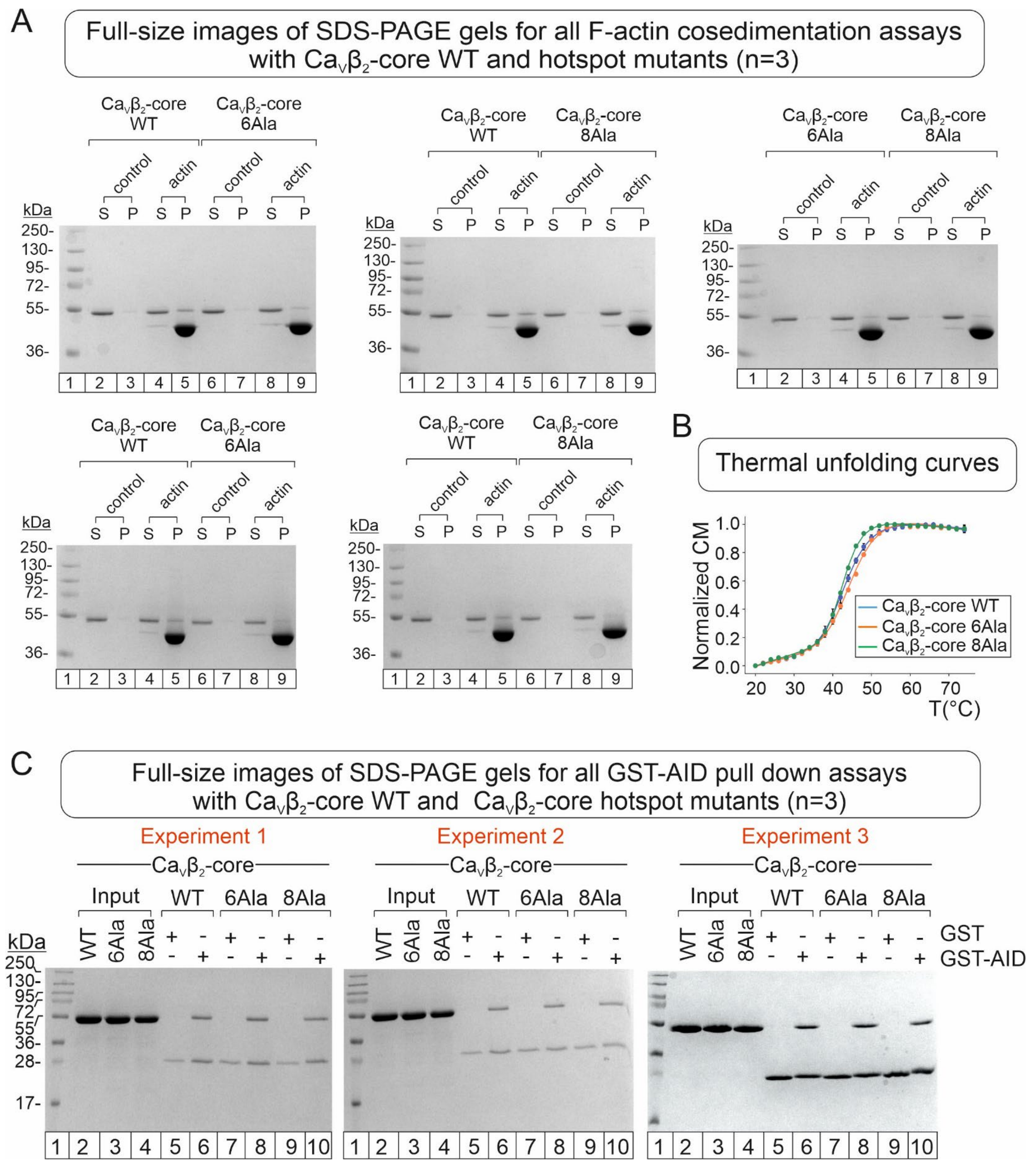

Supplementary Figure 11. F-actin co-sedimentation assays, thermal stability, and GST-AID pull downs of $Ca_V\beta_2$-core, and the hotspot mutants, $Ca_V\beta_2$-core 6Ala and $Ca_V\beta_2$-core 8Ala. A, Full-size images of the SDS-PAGE for all F-actin cosedimentation assays with $Ca_V\beta_2$-core wild-type (WT), $Ca_V\beta_2$-core 6Ala (6Ala) and $Ca_V\beta_2$-core 8Ala (8Ala). For each gel, lane 1 contains the molecular weight standards, S and P denote supernatant and pellet, respectively, and control and actin denote that the assay was performed in the absence (WT and hotspot mutants alone) and presence of actin, respectively. **B**, Thermal unfolding curves for $Ca_V\beta_2$-core WT, 6Ala, and 8Ala with Tm values (± SE, °C) of 43.9± 0.1, 45.6±0.3 and 43.1±0.1, respectively. CM, center of mass, determined as in Eq. 1. **C**, Full-size images of all SDS-PAGE gels for GST-AID pull down assays with $Ca_V\beta_2$-core WT and the hotspot mutants 6Ala and 8Ala. Each pull down assay was repeated three times. For each gel: lanes 2–4, input proteins; and lanes 5–10, eluted fractions for the indicated protein in either control pull downs using GST alone as bait (lanes 5, 7, and 9) or test assays using GST-AID (lanes 6, 8, and 10). The same molecular weight standards were used in each experiment, with the molecular masses in kDa shown adjacent to the left-most gel.

Supplementary Figure 12

```
beta-2      ---------------------------------MQCCGLVHRRRVRVSRGSADSYTSRPS   27
beta-4      MSSSSYAKNGTADGPHSPTSQVARGTTTRRSRLKRSDGSTTSTSFILRQGSADSYTSRPS   60
                                              :. * .    . : :***********

beta-2      DSDVSLEEDREAVRREAERQAQAQLEKAKTKPVAFAVRTNVRYSAAQEDDVPVPGMAISF   87
beta-4      DSDVSLEEDREAIRQEREQQAAIQLERAKSKPVAFAVKTNVSYCGALDEDVPVPSTAISF   120
            ************:*:* *:**  ***:**:*******:*** *..* ::*****. ****

beta-2      EAKDFLHVKEKFNNDWWIGRLVKEGCEIGFIPSPVKLENMRLQHEQRAKQGKFYSSKSGG   147
beta-4      DAKDFLHIKEKYNNDWWIGRLVKEGCEIGFIPSPLRLENIRIQQEQ--KRGRFHGGKSSG   178
            :******:***:**********************::***:*:*:**  *:*:*:..**.*

beta-2      NSSSSLGDIVPSSRKSTPPSSAIDIDATGLDAEENDIPANHRSPKPSANSVTSPHSKEKR   207
beta-4      NSSSSLGEMVSGTFRATPTSTAK-------------------------------------   201
            *******::* .: ::** *:*

beta-2      MPFFKKTEHTPPYDVVPSMRPVVLVGPSLKGYEVTDMMQKALFDFLKHRFEGRISITRVT   267
beta-4      -QKQKVTEHIPPYDVVPSMRPVVLVGPSLKGYEVTDMMQKALFDFLKHRFDGRISITRVT   260
                * *** ****************************************:*********

beta-2      ADISLAKRSVLNNPSKHAIIERSNTRSSLAEVQSEIERIFELARTLQLVVLDADTINHPA   327
beta-4      ADISLAKRSVLNNPSKRAIIERSNTRSSLAEVQSEIERIFELARSLQLVVLDADTINHPA   320
            ****************:***************************:***************

                                *          *     *     *
beta-2      QLSKTSLAPIIVYVKISSPKVLQRLIKSRGKSQAKHLNVQMVAADKLAQCPPQESFDVIL   387
beta-4      QLIKTSLAPIIVHVKVSSPKVLQRLIKSRGKSQSKHLNVQLVAADKLAQCPP-EMFDVIL   379
            ** *********:**:*****************:******:*********** * *****

beta-2      DENQLEDACEHLADYLEAYWKATHPPSSNLPNPLLSRTLATSTLPLSPTLASNSQGSQGD   447
beta-4      DENQLEDACEHLGEYLEAYWRATHTTSSTPMTPLLGRNLGSTALSPYPTAISGLQSQRMR   439
            ************.:******:***  **.  .***.*.*.:::*   **  *. *..:

beta-2      QRTDRSAPRSASQAEEEPCLEPVKKSQHRSSSATHQNHRSGTGRGLSRQETFDSETQESR   507
beta-4      HS-NHS-------TENS----PIERRSLMTSDENYHNERAR-----KSRNRLSSSSQHS-   481
            :  ::*       :*:.    *::: .  :*. .::*.*:      . :: :.*.:*.*

beta-2      DSAYVEPKEDYSHEHVDRYVPHREHNHREESHSSNGHRHREPRHRTRDMGRDQDHNECSK   567
beta-4      RDHYPLVEEDYPDSYQDTYKPHRNRGSPGGYSH-------DSRHRL--------------   520
             . *   :***..:  * * ***::.              : ***

beta-2      QRSRHKSKDRYCDKEGEVISKRRSEAGEWNRDVYIRQ   604
beta-4      -------------------------------------   520
```

Supplementary Figure 12. Sequence alignment of Ca$_{V}\beta_{2}$ and Ca$_{V}\beta_{4}$. The amino acid sequences of Ca$_{V}\beta_{2}$ (UniProtKB Q8VGC3-2) and Ca$_{V}\beta_{4}$ (UniProtKB O00305-1) used in this study were aligned using Clustal Omega. The SH3 and GK domains are enclosed in pink and blue, respectively. All four lysine residues involved in the set of self-consistent inter-molecular cross-links identified in Ca$_{V}\beta_{2}$ are fully conserved in Ca$_{V}\beta_{4}$ (shown enclosed in a red frame and labeled with a red asterisk).

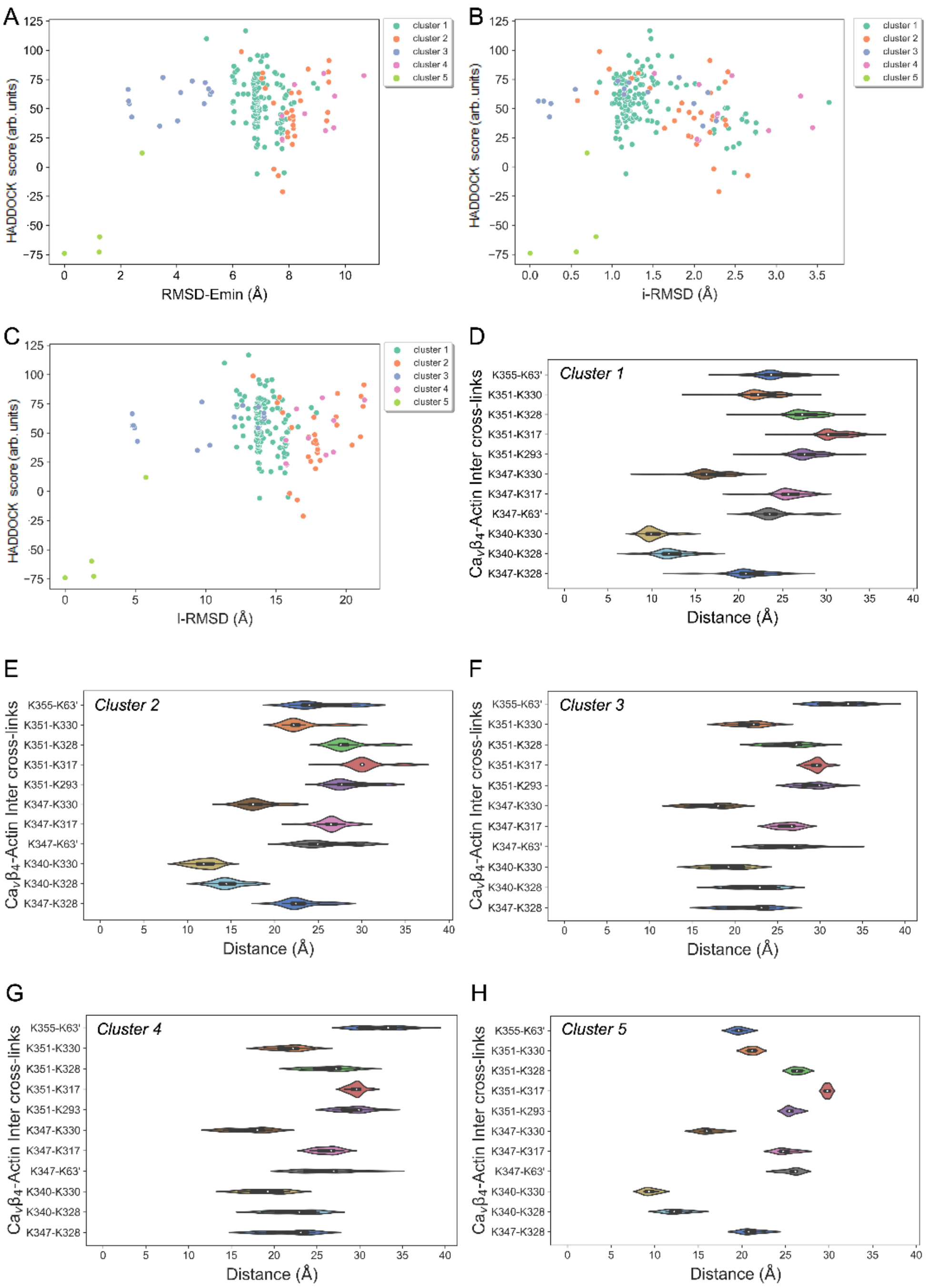

Supplementary Figure 13. Model docking data for Ca$_v$β$_4$ and actin. A, HADDOCK score versus global RMSD (RMSD-Emin) plot for Ca$_v$β$_4$–actin docking structures. **B**, HADDOCK score versus interface RMSD (i-RMSD) plot for Ca$_v$β$_4$–actin docking structures. **C**, HADDOCK score versus ligand RMSD (l-RMSD) plot for Ca$_v$β$_4$–actin docking structures. **D-H**, Mapped Euclidean Cα-Cα cross-linked pair distance distribution for all the docking structures belonging to the clusters 1-5, respectively, generated by HADDOCK 2.4 for Ca$_v$β$_4$–actin. The three RMSD metrics in panels A-C are calculated as explained in Supplementary Figure 4; the reference structure for all RMSD calculations is the one with the lowest HADDOCK score, which in the Ca$_v$β$_4$–actin docking belongs to cluster 5. The cluster size is given in Supplementary Table 4B.

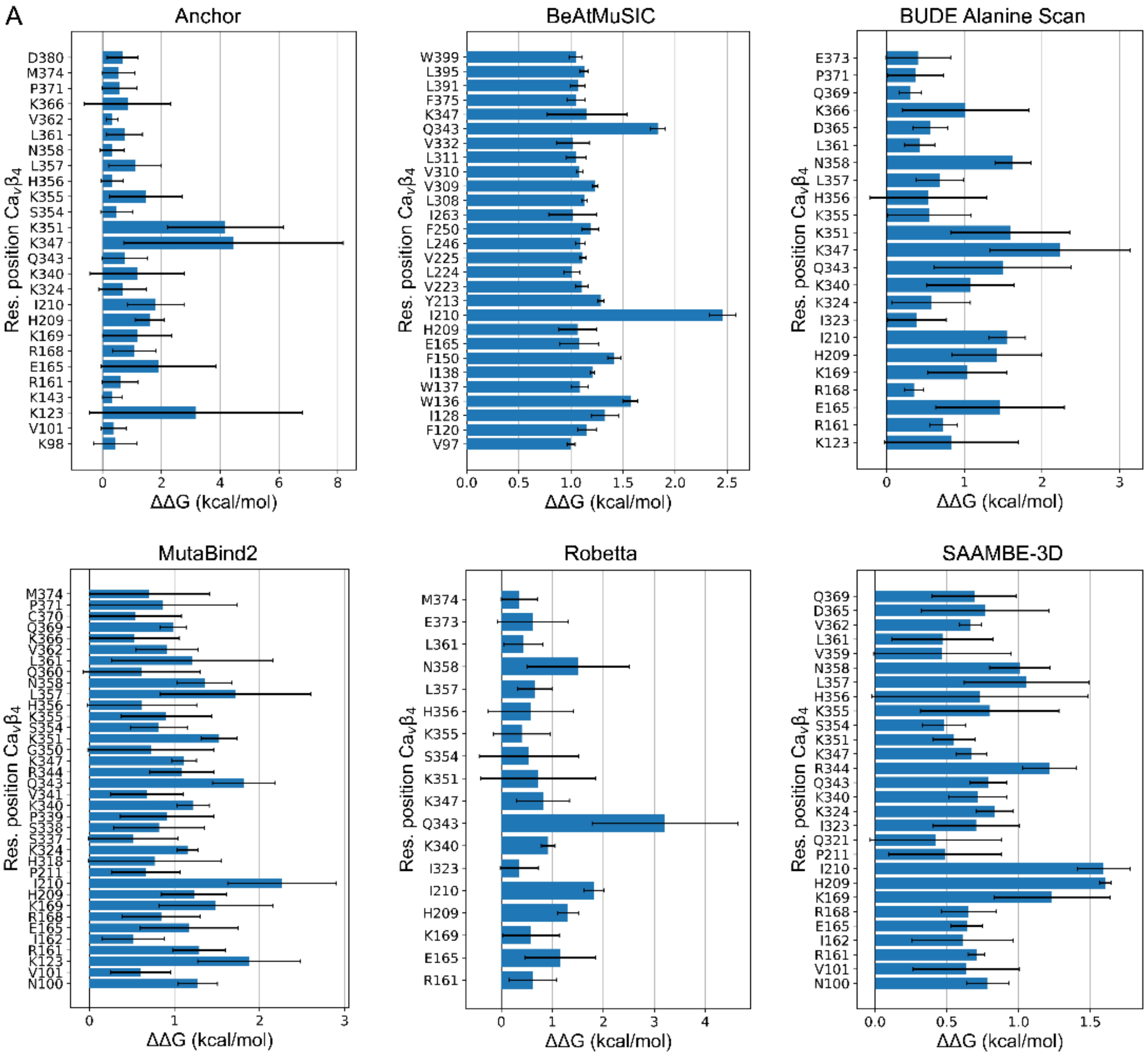

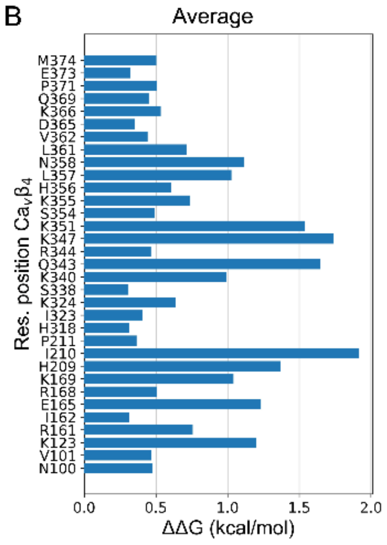

Supplementary Figure 14. Computational alanine scanning analysis to identify hotspot residues on $Ca_V\beta_4$ at its actin interaction interface. **A**, $Ca_V\beta_4$ actin interface hotspots were predicted as described in Supplementary Figure 9 using the six webservers indicated. Changes in binding free energy upon alanine mutation (ΔΔG) calculated with the six predictors were averaged across the top four docking models of the selected cluster. Error bars correspond to the standard deviation. **B**, ΔΔG values averaged over all six predictors. Only mutations resulting in a ΔΔG higher than 0.3 kcal/mol are shown.

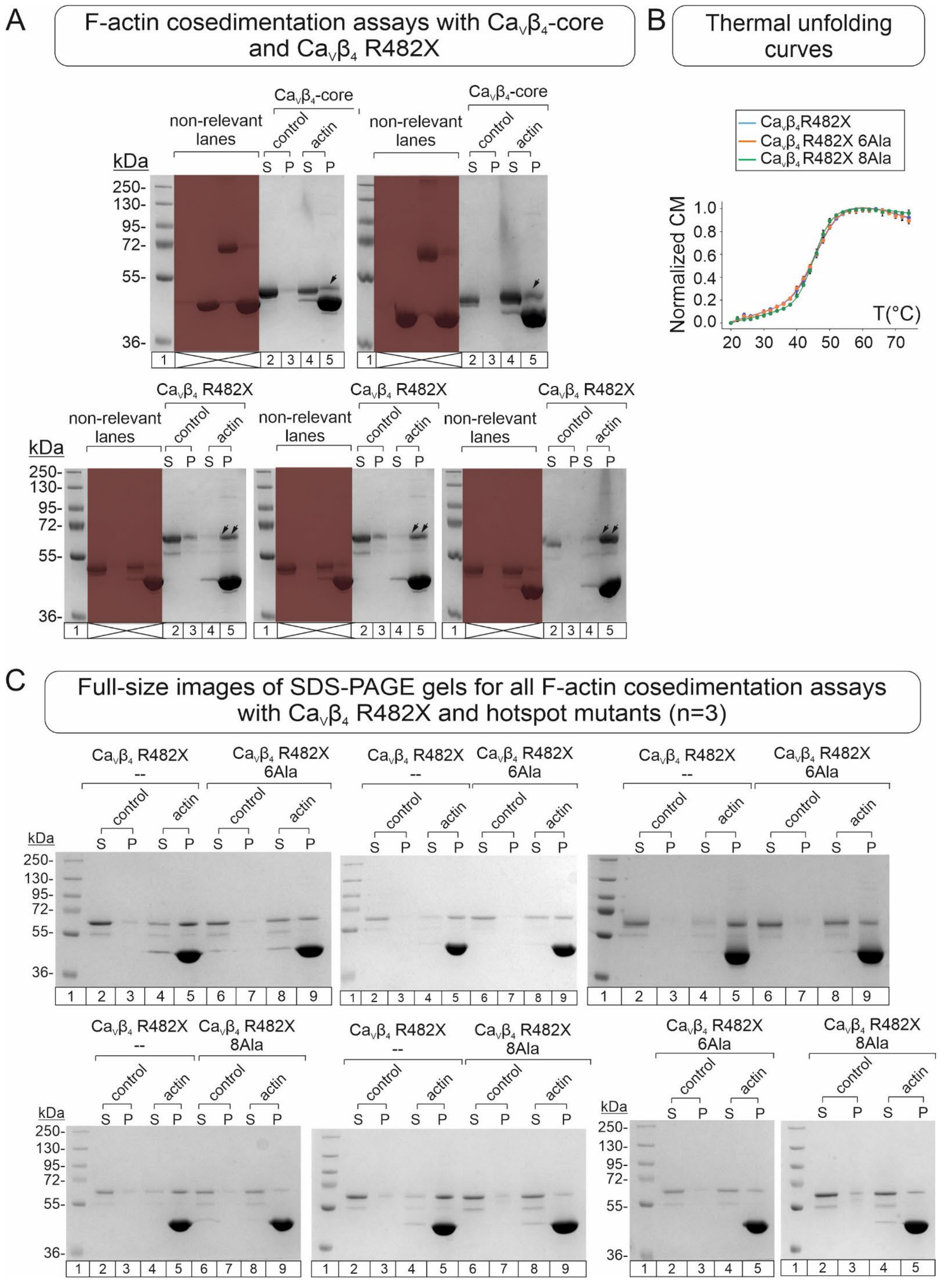

Supplementary Figure 15. Comparison of thermal stability and binding to F-actin and GST-AID of Ca$_V$β$_4$ R482X and the hotspot mutants. A, Full-size images of the SDS-PAGE of separate F-actin cosedimentation assays with Ca$_V$β$_4$-core and Ca$_V$β$_4$ R482X. For each gel: lane 1, molecular weight standards and lanes 2, 3, 4 and 5 correspond to supernatant (S) and pellet (P) denote and pellet fractions

for the indicated assay. Control and actin denote the assay performed in the absence and presence of actin, respectively. Lanes relevant for this study are enclosed in a black square while irrelevant lanes outside the scope of this work are not numbered and marked with a blue cross in the gel. **B**, Thermal unfolding curves for $Ca_V\beta_4$ R482X bearing no alanine substitution, $Ca_V\beta_4$ R482X 6Ala, and $Ca_V\beta_4$ R482X 8Ala with Tm values (± SE, °C) of 47.7±0.2, 47.7±0.3 and 45.9±0.2, respectively. CM, center of mass, as determined as in Eq. 1. **C**, Full-size images of the SDS-PAGE of all F-actin cosedimentation assays with $Ca_V\beta_4$ R482X bearing no Ala substitutions (--), $Ca_V\beta_4$ R482X 6Ala, and $Ca_V\beta_4$ R482X 8Ala. For each gel: lane 1, molecular weight standards; S and P denote supernatant and pellet, respectively; and control and actin denote the assay performed in the absence ($Ca_V\beta_4$ R482X and hotspot mutants alone) and presence of actin, respectively. The same molecular weight standards were used in each experiment, with the molecular masses in kDa shown adjacent to the left-most gel.

Supplementary Figure 15D

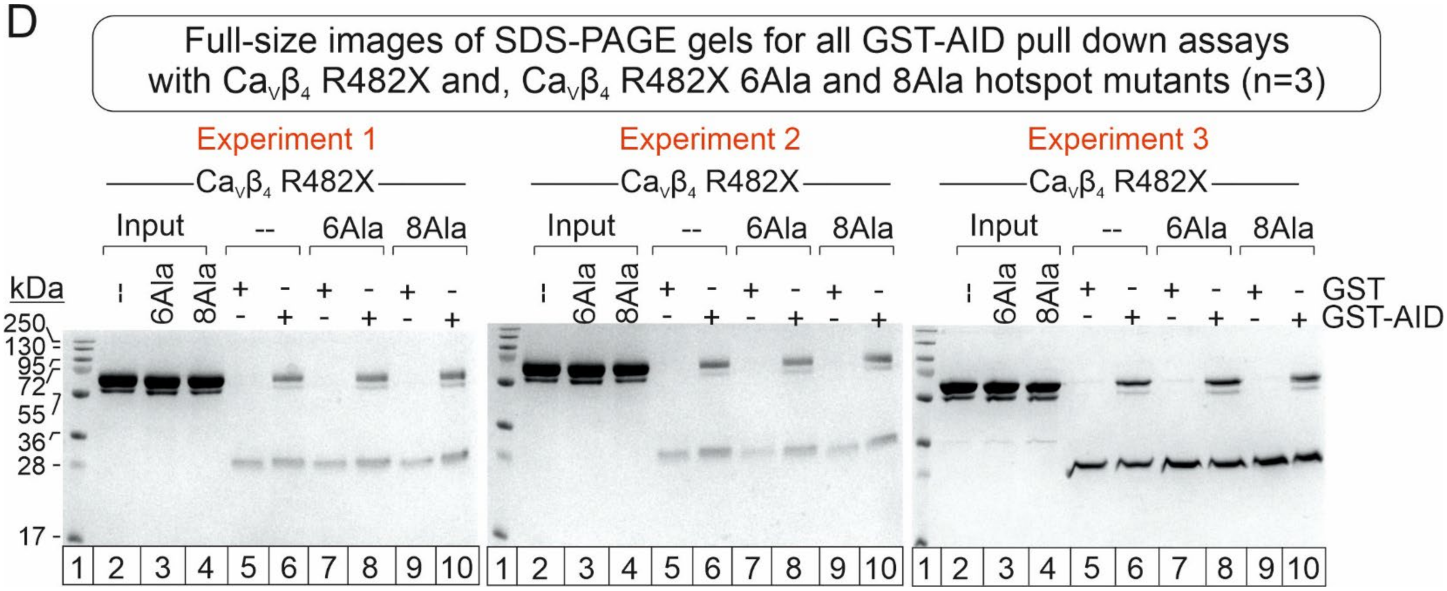

Supplementary Figure 15. Comparison of thermal stability and binding to F-actin and GST-AID of $Ca_V\beta_4$ R482X and the hotspot mutants. D, Full-size images of all SDS-PAGE gels for GST-AID pull down assays with $Ca_V\beta_4$ R482X with no Ala substitutions (--), $Ca_V\beta_4$ R482X 6Ala (6Ala), and $Ca_V\beta_4$ R482X 8Ala (8Ala). Each pull down assay was repeated three times. For each gel: lane 1, molecular weight standards, lanes 2–4, input proteins; and lanes 5–10, eluted fractions for the indicated protein in either control pull downs using GST alone as bait (lanes 5, 7, and 9) or test assays using GST-AID (lanes 6, 8, and 10). The same molecular weight standards were used in each experiment, with the molecular masses in kDa shown adjacent to the left-most gel.

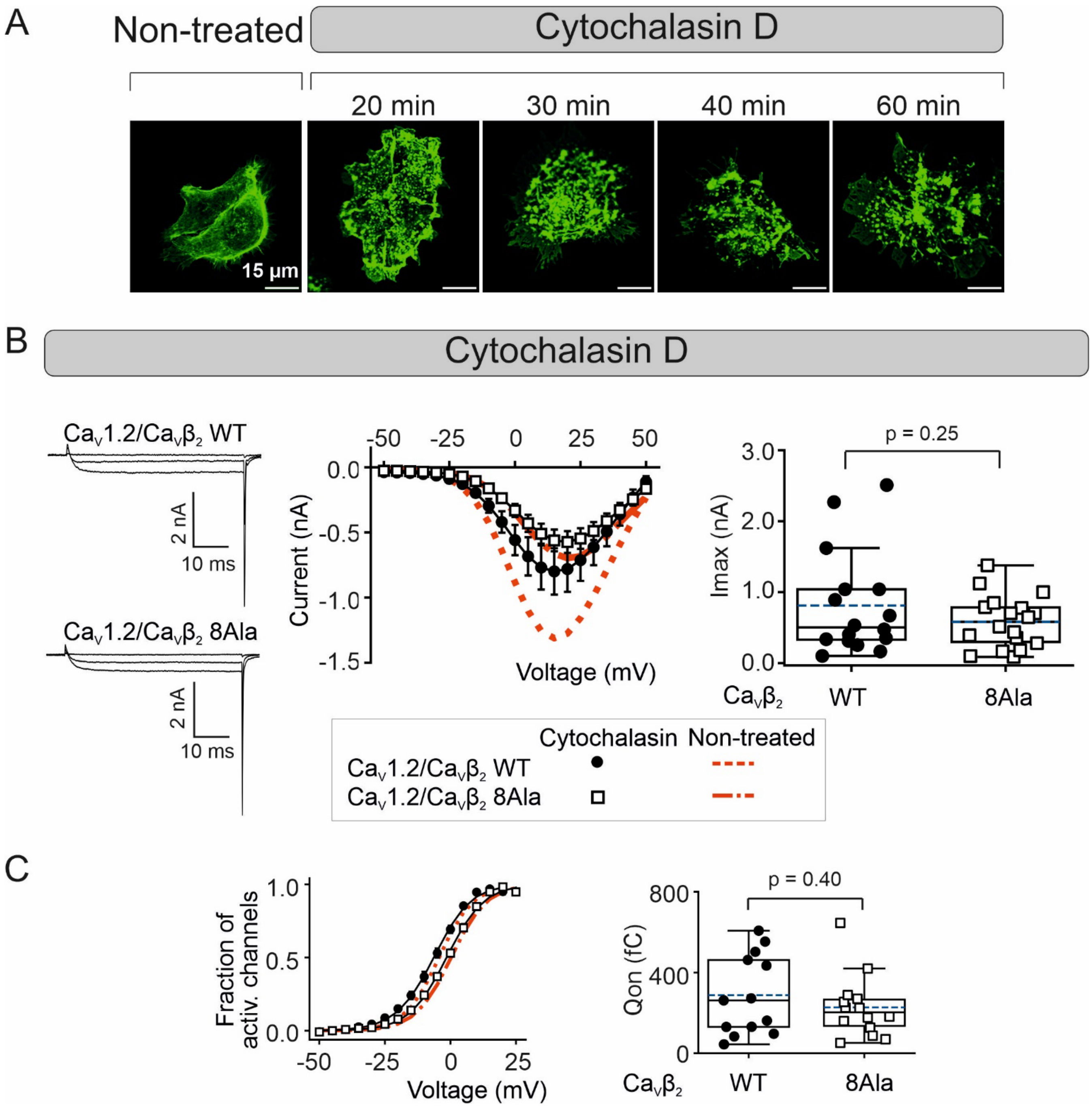

Supplementary Figure 16. Pharmacological disruption of actin filaments inhibits current reduction mediated by the actin-association-deficient $Ca_V\beta_2$ mutant. **A**, Representative laser scanning confocal images of HEK293 cells stained for actin using phalloidin-488 after incubation with 10 µM of the actin filament disrupter cytochalasin D for the indicated time periods. The experiment was repeated twice with cells from different number of passages. **B**, Representative ionic current traces from cells transiently cotransfected with $Ca_V1.2$ (fused to mNeonGreen) and either $Ca_V\beta_2$ WT or $Ca_V\beta_2$ 8Ala (fused to mRFP) (left panel) and plot of the mean current amplitude at voltages between -50 to +50 mV in 5 mV increments from a holding potential of -90 mV (middle panel). Recordings were done after incubation for 40 minutes with 10 µM of cytochalasin D. Only the current traces induced by -40, +15, and +40 mV pulses are shown. Data are presented as mean ± SEM. Imax: -0.81 ± 0.18 nA for $Ca_V1.2/Ca_V\beta_2$ WT (number of individual recorded cells, n = 16) and -0.58 ± 0.08 nA for $Ca_V1.2/Ca_V\beta_2$ 8Ala (n = 18). For comparison the I/V curves obtained from non-cytochalasin D

treated cells expressing the same subunit combinations (from Figure 6, main text) are shown in red lines. The box plot (right panel) shows the average peak current (Imax) for the indicated channel subunit combination taken from the I/V plot. Each dot represents an individual recorded cell. Dashed lines represent the mean and the continuous line the median, the box edges denote the interquartile range (25th–75th percentiles) and whiskers, the 1.5× interquartile range. **C**, Plot of the fraction of activated channels versus voltage and box plot of the total charge movement (Qon) obtained from the cells in panel B. Red lines correspond to the curves obtained for non-cytochalasin D treated cells expressing the same subunit combinations (from Figure 6, main text). Data are presented as mean ± SEM. In the box plot: mean (dashed line), median (continuous line), interquartile range (25th–75th percentiles, box edges) and whiskers (1.5× interquartile range). Each dot represents an individual recorded cell (n). Qon was calculated from the integral of the On gating current during the voltage step to the reversal potential for the carrier ion. Qon (mean ± S.E.M: 288 ± 53 fC (n, = 13) and 227 ± 40 fC (n = 14) for $Ca_V1.2/Ca_V\beta_2$ WT and $Ca_V1.2/Ca_V\beta_2$ 8Ala, respectively. p-values were determined by two-sided t-test.

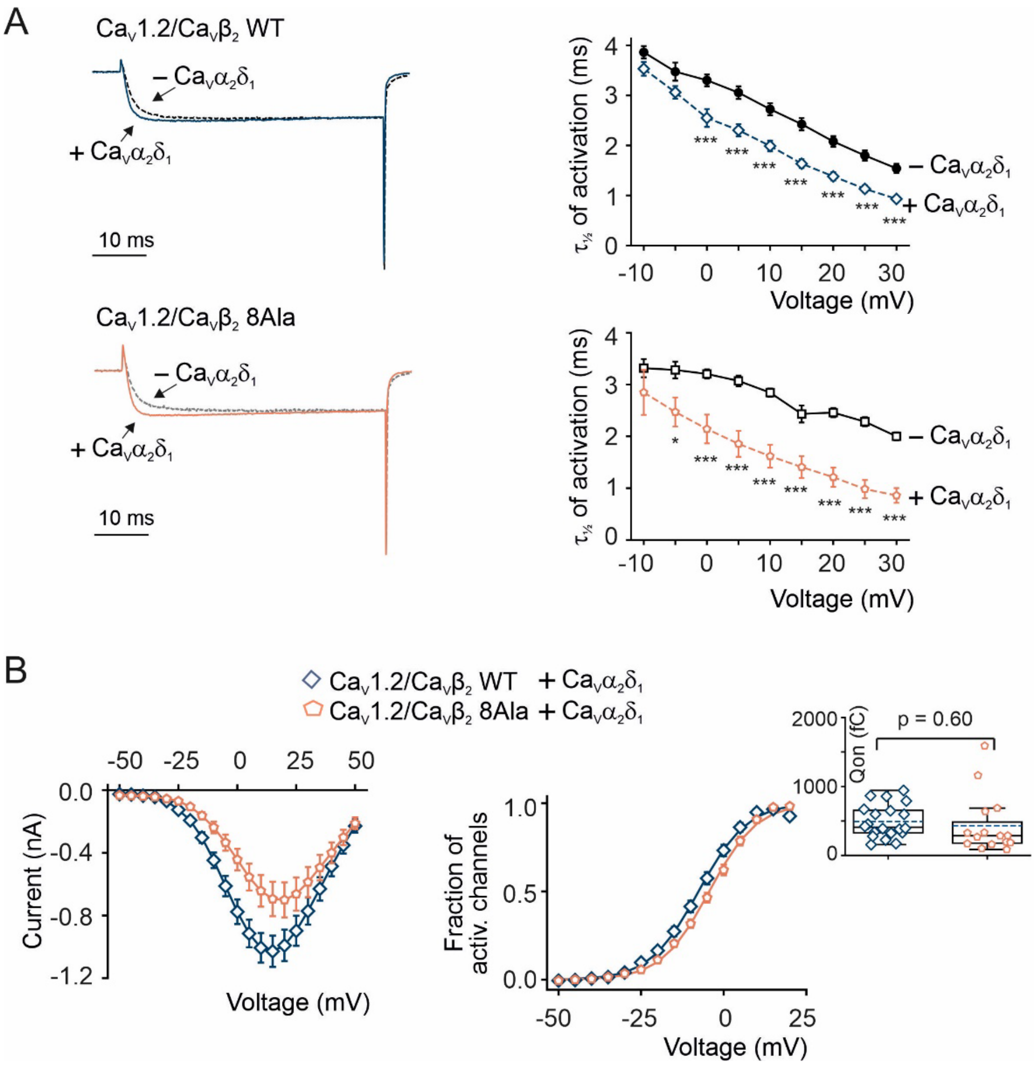

Supplementary Figure 17. Expression of $Ca_V\alpha_2\delta_1$ subunit marginally affects the functional competence of the $Ca_V1.2/Ca_V\beta_2$ core complex in HEK293 cells. **A**, Representative normalized ionic current traces at +20 mV and plot of the mean $\tau_{½}$ values (± S.E.M) for the current activation at different voltages from cells expressing $Ca_V1.2/Ca_V\beta_2$ (either $Ca_V\beta_2$ WT or $Ca_V\beta_2$ 8Ala hotspot mutant) with and without $Ca_V\alpha_2\delta_1$. * $p < 0.05$; ***$p < 0.001$; t-test. $Ca_V\alpha_2\delta_1$ speeds up the activation kinetics. **B**, Current-to-voltage (I/V) plot and fraction of activated channels versus voltage plot obtained from cells expressing $Ca_V1.2/\beta_2/\alpha_2\delta_1$ channels encompassing either $Ca_V\beta_2$ WT (number of recorded cells, n =20) or $Ca_V\beta_2$ 8Ala hotspot mutant (n =15). Ionic currents were elicited by steps to voltages between -50 to +50 mV in 5 mV increments from a holding potential of -90 mV. Data are presented as mean ± SEM. Inset shows the box of the total charge movement (Qon) with mean (dashed line), median (continuous line), interquartile range (25th–75th percentiles, box edges) and whiskers (1.5× interquartile range). Each dot represents an individual recorded cell. Qon was calculated from the integral of the On gating current during the voltage step to the reversal potential for the carrier ion determined empirically by stepping to several potentials in 2 mV increments. Mean values ± S.E.M: Imax, -1.1 ± 0.1 nA and -0.7 ± 0.1 nA (p = 0.03), Qon, 491 ± 52 fC and 432 ± 107 fC for $Ca_V1.2/\beta_2/\alpha_2\delta_1$ encompassing $Ca_V\beta_2$ WT and 8Ala mutant, respectively. P-values were determined by two-sided t-test. ($Ca_V\alpha_2\delta_1$ from Scholl et al., *Nature Genetics* **45**, 1050-4 (2013)).

Supplementary Figure 18

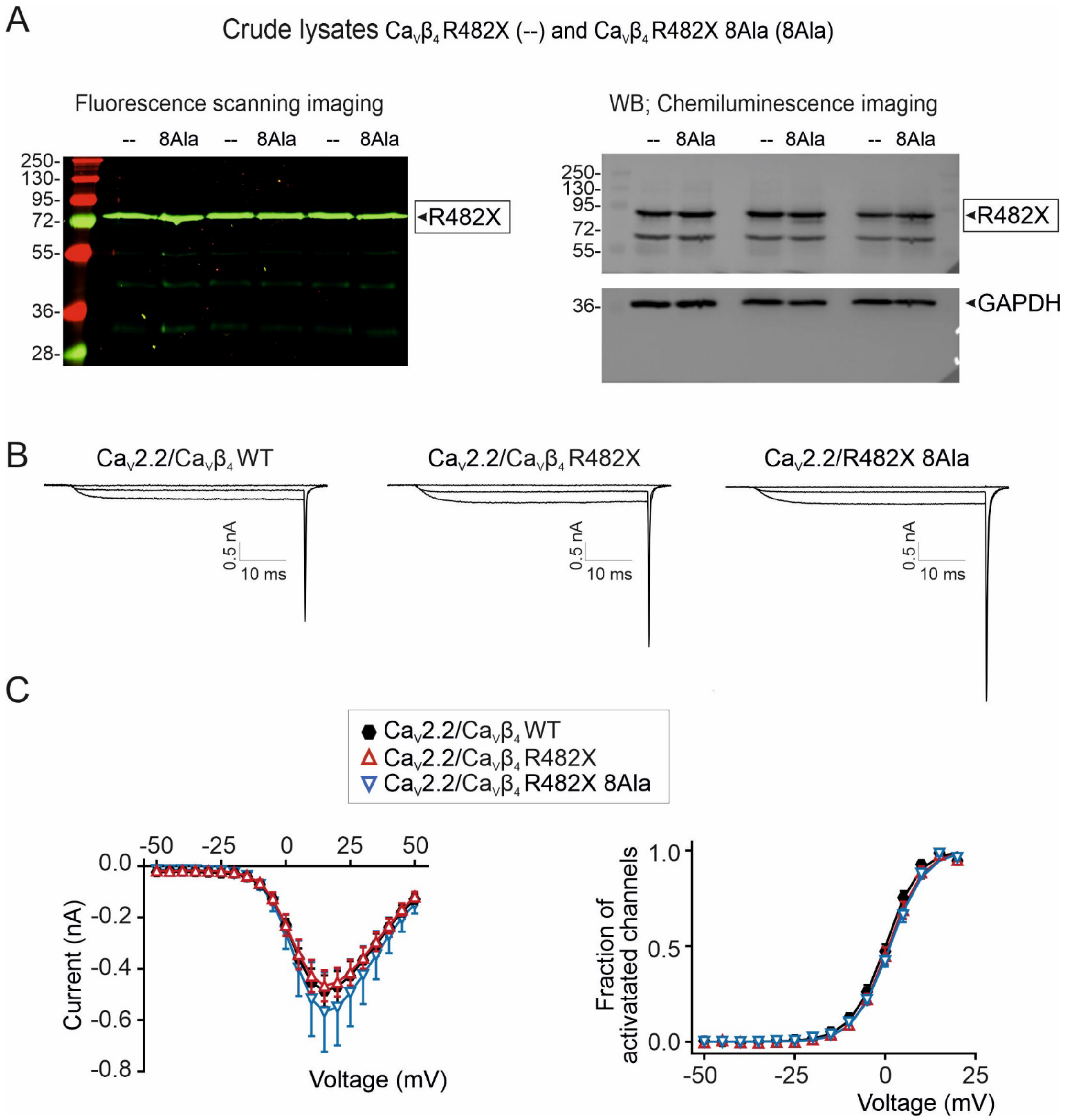

Supplementary Figure 18. Effect of $Ca_V\beta_4$ R482X and the actin-association-deficient $Ca_V\beta_4$ R482X mutant on $Ca_V2.2$ mediated currents in HEK 293 cells. **A**, Full images of SDS-PAGE from crude lysates of HEK293 cells expressing $Ca_V\beta_4$ R482X (--) and $Ca_V\beta_4$ R482X 8Ala (8Ala), both fused to mCherry. Lysates from three separate experiments were loaded in two separate gels for fluorescence scanning (left panel) and for Western blot analysis (WB) with anti-$Ca_V\beta_4$ and anti-GAPDH antibodies. **B**, Representative current traces mediated by heterologous $Ca_V2.2$ coexpressed in HEK293 cells with either wild-type $Ca_V\beta_4$ ($Ca_V\beta_4$ WT) or the $Ca_V\beta_4$ R482X variant bearing ($Ca_V\beta_4$ R482X 8Ala) or not ($Ca_V\beta_4$ R482X) the hotspot mutations leading to impaired association with actin. For visualization of transfected cells, $Ca_V2.2$ was fused to GFP while $Ca_V\beta_4$ constructs to mCherry. Currents were elicited by voltage steps from -50 to +50 in 5 mV increments from a holding potential of -90 mV and using Ba^{2+} as charge carrier. For clarity, only the current traces induced by -35, +20, and +45 mV pulses are shown. The pipette solution contained 135 mM cesium methanesulfonate, 10 mM EGTA, 5 mM CsCl, 1 mM $MgCl_2$, and 10 mM HEPES, adjusted to pH 7.3 with CsOH. The extracellular recording solution contained 140 mM tetraethylammonium-$MeSO_3$, 10 mM $BaCl_2$, and 10 mM HEPES buffer, adjusted to pH 7.3 with tetraethylammonium hydroxide. **C**, Plots of ionic current versus voltage (I/V) and fraction of activated channels versus voltage obtained from HEK293 cells transiently expressing the indicated constructs shown in A. Data are presented as mean ± SEM. Number of recorded cells (n) = 13 for $Ca_V2.2$/$Ca_V\beta_4$ WT, 12 for $Ca_V2.2$/$Ca_V\beta_4$ R482X, and 12 for $Ca_V2.2$/$Ca_V\beta_4$ R482X 8Ala. Mean current amplitudes (± SEM) are shown.

Supplementary Table 1. Summary of the cross-linked spectrum matches (CSMs). The table summarizes the number of CSMs per class (total, inter, intra, looplinked and dead-end) identified by the different software.

Cross-linker	Number of replicates	Software	Total CSMs	Inter CSMs	Intra CSMs	Looplinked CSMs	Dead-end CSMs
DSSO	5	MaxLynx	249	52	24	11	162
		MetaMorpheus	893	179	120	68	526
		Merox	67	14	12	1	40
DSBU	4	MaxLynx	331	14	31	68	218
		MetaMorpheus	370	19	53	80	218
		Merox	103	7	12	14	70

Supplementary Table 2. Intermolecular cross-links between actin and $Ca_V\beta2$ identified with DSSO and DSBU cross-linkers. Only unique intermolecular cross-links (inter-XLs) are reported; for each inter-XL, the replicate (Rep) number and the number of cross-linked spectra matches (CSMs) detected with each software are listed, with ‘x’ indicating ‘not detected’. All CSMs fulfill the false discovery rate cutoff of 0.01.

Unique inter-XLs (ID)	DSSO[a]	DSBU[a]	Sequence Actin	Position	Sequence $Ca_V\beta_2$	Position recombinant protein ß2-core	Corresponding position Uniprot Q8VGC3-2	DSSO Rep_1 (CSMs) ML_MM_Me	DSSO Rep_2 (CSMs) ML_MM_Me	DSSO Rep_3 (CSMs) ML_MM_Me	DSSO Rep4 (CSMs) ML_MM_Me	DSSO Rep5 (CSMs) ML_MM_Me	DSBU Rep1 (CSMs) ML_MM_Me	DSBU Rep2 (CSMs) ML_MM_Me	DSBU Rep3 (CSMs) ML_MM_Me	DSBU Rep4 (CSMs) ML_MM_Me	Selected unique inter-XLs[b]	Self consistent inter-XLs[c]
1	*	*	HQGVMVGMGQ**K**DSYVGDEAQSK	52	LI**K**SR	364	354	2_3_1	0_19_0	0_1_0	0_1_0	0_1_0	1_1_0	x	x	x	*	
2	*		HQGVMVGMGQ**K**DSYVGDEAQSK	52	G**K**SQAK	368	358	0_1_0	x	x	0_1_0	x	x	x	x	x		
3	*		DSYVGDEAQS**K**R	63	A**K**QGK	146	136	0_1_0	x	x	0_3_0	x	x	x	x	x		
4	*		DSYVGDEAQS**K**R	63	ISSP**K**VLQR	357	347	0_6_0	x	x	0_2_0	x	x	x	x	x		
5	*	*	DSYVGDEAQS**K**R	63	LI**K**SR	364	354	6_7_2	1_1_0	x	1_2_1	0_1_0	2_1_1	x	1_1_0	x	*	*
6	*		DSYVGDEAQS**K**R	63	G**K**SQAK	368	358	0_2_0	x	x	0_1_0	x	x	x	x	x		
7	*		DSYVGDEAQS**K**R	63	SQA**K**HLNVQMVAADK	372	362	2_5_0	x	x	x	x	x	x	x	x	*	*
8	*		**K**DLYANNVMSGGTTMYPGIADR	293	G**K**SQAK	368	358	2_1_0	x	x	0_1_0	x	x	x	x	x	*	*
9	*		MQ**K**EITALAPSTMK	317	A**K**QGK	146	136	1_1_0	x	x	x	x	x	x	x	x	*	
10	*		MQ**K**EITALAPSTMK	317	ISSP**K**VLQR	357	347	0_2_0	0_2_0	x	0_1_0	x	x	x	x	x		
11	*		MQ**K**EITALAPSTMK	317	LI**K**SR	364	354	2_3_1	5_5_0	x	3_1_2	x	x	x	x	x	*	*
12	*		MQ**K**EITALAPSTMK	317	G**K**SQAK	368	358	6_12_0	0_1_0	x	3_5_0	x	x	x	x	x	*	*
13	*		EITALAPSTM**K**IK	328	A**K**QGK	146	136	2_1_0	x	x	0_1_0	x	x	x	x	x	*	
14		*	EITALAPSTM**K**IK	328	ISSP**K**VLQR	357	347	x	x	x	x	x	0_1_1	x	0_1_0	x	*	*
15	*	*	EITALAPSTM**K**IK	328	LI**K**SR	364	354	1_3_1	1_2_0	x	x	x	1_1_0	x	x	x	*	*
16	*		EITALAPSTM**K**IK	328	G**K**SQAK	368	358	3_4_1	x	x	1_3_0	x	x	x	x	x	*	*
17	*		I**K**IIAPPER	330	A**K**QGK	146	136	3_7_0	1_12_0	x	1_4_0	x	x	x	x	x	*	
18	*	*	I**K**IIAPPER	330	VTADISLA**K**R	284	274	x	x	x	x	0_1_0	x	1_0_1	1_1_0	x	*	
19	*	*	I**K**IIAPPER	330	ISSP**K**VLQR	357	347	0_12_0	1_12_1	0_2_0	0_3_0	0_1_0	2_2_1	1_1_0	2_2_1	1_1_0	*	*
20	*	*	I**K**IIAPPER	330	LI**K**SR	364	354	0_4_0	3_10_0	0_5_0	1_9_0	0_1_0	0_3_1	x	1_2_1	x	*	*
21	*		I**K**IIAPPER	330	G**K**SQAK	368	358	0_5_2	0_11_0	0_3_0	0_5_0	0_1_0	x	x	x	x	*	*
22	*	*	I**K**IIAPPER	330	SQA**K**HLNVQMVAADK	372	362	x	0_4_0	x	x	x	x	x	0_1_0	x		

[a] Unique inter-XLs detected with the indicated cross-linker.
[b] Inter-XLs were selected when detected with more than one software (MaxLynx (ML), Metamorpheus (MM) and Merox (Me)).
[c] Inter-XLs were identified as self-consistent by DisVis.

Supplementary Table 3. Data consistency and violation analysis of XL-MS data obtained for Ca$_V$β$_2$-actin interaction performed with DisVis webserver. Only self-consistent restraints are listed. Note that the restraints are defined as fixed chain/scanning chain (actin-Ca$_V$β$_2$), i.e swapped order compared to the main text.

(A) Ca$_V$β$_2$–actin dimer			
**#**	**Restraint**	**Average violated fraction**	**Standard deviation**
1	K328-K354	0.16	0.20
2	K328-K358	0.17	0.19
3	K330-K354	0.17	0.21
4	K330-K358	0.18	0.19
5	K293-K358	0.20	0.18
6	K317-K354	0.23	0.17
7	K317-K358	0.24	0.16
8	K328-K347	0.27	0.19
9	K330-K347	0.29	0.19
10	K63'-K354	0.42	0.18
11	K63'-K362	0.43	0.18

(B) Ca$_V$β$_2$–actin monomer			
**#**	**Restraint**	**Average violated fraction**	**Standard deviation**
1	K330-K354	0.15	0.23
2	K328-K354	0.16	0.23
3	K328-K358	0.18	0.23
4	K330-K358	0.18	0.23
5	K317-K354	0.20	0.22
6	K317-K358	0.22	0.22
7	K293-K358	0.23	0.21
8	K330-K347	0.25	0.22
9	K328-K347	0.25	0.21
10	K63-K362	0.74	0.34
11	K63-K354	0.78	0.30

Supplementary Table 4. Statistics from the HADDOCK information-driven docking calculations for the $Ca_V\beta_2$–actin and $Ca_V\beta_4$–actin complex models.

**A**, HADDOCK statistics for **$Ca_V\beta_2$–actin dimer** docking structures								
**Cluster**	**HADDOCK score (arb. units)**	**Cluster size**	**Global RMSD (Å)**	**van der Waals energy (E_{vdw}, kcal^{-1} mol)**	**Electrostatic energy (E_{elec}, kcal^{-1} mol)**	**Desolvation energy (E_{desol}, kcal^{-1} mol)**	**Restraints violation energy (E_{air}, kcal^{-1} mol)**	**Buried surface area (BSA, Å^2)**
#1	-67.8 ± 7.0	198	1.4 ± 1.1	-87.7 ± 9.1	-525.3 ± 104.4	43.3 ± 6.0	815.8 ± 55.5	3208.0 ± 203.6

**B**, HADDOCK statistics for **$Ca_V\beta_4$–actin dimer** docking structures								
**Cluster**	**HADDOCK score (arb. units)**	**Cluster size**	**Global RMSD (Å)**	**van der Waals energy (E_{vdw}, kcal^{-1} mol)**	**Electrostatic energy (E_{elec}, kcal^{-1} mol)**	**Desolvation energy (E_{desol}, kcal^{-1} mol)**	**Restraints violation energy (E_{air}, kcal^{-1} mol)**	**Buried surface area (BSA, Å^2)**
#1	5.5 ± 10.9	135	7.4 ± 0.4	-68.8 ± 1.2	-328.3 ± 59.0	18.4 ± 8.1	1216.8 ± 196.3	2795.9 ± 230.3
#2	-2.7 ± 14.6	29	7.7 ± 0.2	-108.0 ± 12.4	-301.1 ± 20.5	22.7 ± 6.2	1428.7 ± 50.0	3540.4 ± 338.7
#3	43.0 ± 7.1	15	3.0 ± 0.7	-47.9 ± 5.1	-402.8 ± 59.8	30.5 ± 4.9	1409.0 ± 104.7	2128.5 ± 91.3
#4	27.9 ± 4.6	10	8.6 ± 0.8	-65.2 ± 11.1	-254.7 ± 61.2	10.8 ± 0.8	1332.1 ± 161.0	2294.1 ± 410.6
#5	-48.6 ± 35.4	4	1.3 ± 1.0	-94.2 ± 17.7	-512.6 ± 103.4	34.3 ± 2.9	1138.5 ± 114.0	3424.7 ± 481.7

**C**, HADDOCK statistics for **$Ca_V\beta_2$–actin monomer** docking structures								
**Cluster**	**HADDOCK score (arb. units)**	**Cluster size**	**Global RMSD (Å)**	**van der Waals energy (E_{vdw}, kcal^{-1} mol)**	**Electrostatic energy (E_{elec}, kcal^{-1} mol)**	**Desolvation energy (E_{desol}, kcal^{-1} mol)**	**Restraints violation energy (E_{air}, kcal^{-1} mol)**	**Buried surface area (BSA, Å^2)**
#1	-40.5 ± 10.1	57	2.3 ± 1.4	-91.1 ± 6.3	-357.1 ± 58.3	16.1 ± 2.4	1059.7 ± 97.6	2982.6 ± 221.4
#2	3.5 ± 8.6	54	18.5 ± 0.5	-49.0 ± 9.7	-264.7 ± 89.4	7.9 ± 5.6	975.5 ± 104.2	1629.6 ± 200.1
#3	6.1 ± 7.7	25	19.9 ± 1.2	-62.1 ± 5.4	-261.5 ± 41.9	8.6 ± 2.0	1118.6 ± 93.0	1825.1 ± 78.1
#4	2.5 ± 7.3	18	20.2 ± 0.2	-46.4 ± 8.7	-244.3 ± 16.6	11.5 ± 3.6	862.8 ± 51.7	1524.7 ± 36.5
#5	-2.7 ± 3.3	13	9.0 ± 0.7	-63.1 ± 6.7	-469.8 ± 70.3	26.4 ± 6.1	1278.8 ± 57.4	2453.9 ± 184.4
#6	19.7 ± 4.5	10	21.7 ± 0.5	-39.0 ± 5.2	-297.9 ± 56.7	13.3 ± 3.8	1049.5 ± 64.8	1427.1 ± 83.1
#7	14.8 ± 17.5	6	21.0 ± 0.7	-46.2 ± 10.4	-316.8 ± 65.1	14.4 ± 4.1	1099.4 ± 90.2	1621.7 ± 163.9
#8	34.3 ± 6.4	5	21.5 ± 0.1	-36.5 ± 4.8	-210.8 ± 22.8	2.1 ± 1.3	1109.4 ± 157.7	1256.2 ± 107.2

Computational docking was performed on the HADDOCK 2.4 webserver. The resulting docking structures (199, 198 and 188 for the $Ca_V\beta_2$–actin dimer, $Ca_V\beta_4$–actin dimer and $Ca_V\beta_2$–actin monomer complexes, respectively) were clustered based on the interface ligand RMSD (i-l-RMSD) from the lowest HADDOCK score structure using a 7.5 Å cutoff (see Supplementary Table 5). Clusters are ranked by the average HADDOCK score of the top four structures. Global RMSD, intermolecular van der Waals, electrostatic, desolvation and restraints violation energy terms, as well as buried surface area, are also averaged over the top four structures of each cluster.

Supplementary Table 5. Dependence of the cluster metrics on the i-l-RMSD cutoff used for clustering. For each cutoff value, the average and standard deviation of three different RMSD metrics (i-l-RMSD, i-RMSD and l-RMSD, see Methods) are given to assess the similarity among the structures belonging to the first cluster. For all RMSD calculations described below, the reference structure is the model with the lowest HADDOCK score, which belongs to the first cluster for the $Ca_V\beta_2$-actin dimer docking or to the fifth cluster for the $Ca_V\beta_4$-actin dimer docking.

(A) $Ca_V\beta_2$–actin dimer models, cluster #1				
**i-l-RMSD cutoff (Å)**	**cluster size**	**i-l-RMSD (Å)**	**i-RMSD (Å)**	**l-RMSD (Å)**
5.0	181	3.0±1.3	0.9±0.4	3.1±1.6
5.5	184	3.0±1.3	0.9±0.4	3.2±1.6
6.0	184	3.0±1.3	0.9±0.4	3.2±1.6
6.5	185	3.1±1.4	0.9±0.4	3.2±1.7
7.0	186	3.1±1.5	0.9±0.4	3.3±1.8
7.5*	198	3.7±2.6	1.0±0.4	4.3±4.1
8.0	200	3.7±2.6	1.0±0.4	4.3±4.1
8.5	200	3.7±2.6	1.0±0.4	4.3±4.1
9.0	200	3.7±2.6	1.0±0.4	4.3±4.1
9.5	200	3.7±2.6	1.0±0.4	4.3±4.1
10.0	200	3.7±2.6	1.0±0.4	4.3±4.1

* For the top 4 models of the first cluster identified with a 7.5 Å i-l-RMSD cutoff, the i-l-RMSD, i-RMSD and l-RMSD values are 3.1±2.7 Å, 0.9±0.6 Å and 2.8±2.4 Å, respectively.

(B) $Ca_V\beta_4$–actin dimer models, cluster #1				
**i-l-RMSD cutoff (Å)**	**cluster size**	**i-l-RMSD (Å)**	**i-RMSD (Å)**	**l-RMSD (Å)**
5.0	102	15.8±0.9	1.2±0.2	14.0±0.7
5.5	108	15.6±1.1	1.3±0.2	14.1±0.8
6.0	114	15.5±1.4	1.3±0.2	14.0±0.9
6.5	117	15.5±1.5	1.3±0.2	14.1±1.0
7.0	120	15.5±1.6	1.3±0.3	14.1±1.1
7.5*	135	15.6±1.5	1.4±0.5	14.2±1.1
8.0	141	15.6±1.5	1.5±0.5	14.3±1.2
8.5	152	15.2±1.6	1.5±0.5	14.7±1.8
9.0	167	15.3±1.6	1.5±0.5	14.8±1.8
9.5	170	15.3±1.6	1.5±0.5	14.9±1.8
10.0	171	15.3±1.6	1.5±0.5	14.9±1.9

* For the top 4 models of the first cluster identified with a 7.5 Å i-l-RMSD cutoff, the i-l-RMSD, i-RMSD and l-RMSD values are 16.3±0.6 Å, 1.8±0.7 Å and 15.0±0.8 Å, respectively.

(C) $Ca_V\beta_4$–actin dimer models, cluster #2				
**i-l-RMSD cutoff (Å)**	**cluster size**	**i-l-RMSD (Å)**	**i-RMSD (Å)**	**l-RMSD (Å)**
5.0	24	13.7±0.7	2.0±0.4	17.1±1.8
5.5	33	13.1±1.0	1.9±0.5	16.0±2.4
6.0	22	14.0±0.9	2.0±0.5	18.1±1.6
6.5	22	14.0±0.9	2.0±0.5	18.3±1.5
7.0	22	14.0±0.9	2.0±0.5	18.3±1.5
7.5*	29	13.9±1.2	1.9±0.5	17.9±1.9
8.0	30	13.9±1.2	1.9±0.5	17.8±1.9

* For the top 4 models of the second cluster identified with a 7.5 Å i-l-RMSD cutoff, the i-l-RMSD, i-RMSD and l-RMSD values are 13.4±0.3 Å, 2.3±0.2 Å and 16.8±0.7 Å, respectively.

N.B. The cluster analyzed for each of the i-l-RMSD cutoff values listed above is the one containing the top 4 structures of the second cluster identified with a 7.5 Å i-l-RMSD cutoff. For cutoff values 5.0, 7.5 and 8.0 Å, the corresponding cluster is #2 (as shown in the Supplementary Table 5C header), whereas for the cutoff range 5.5-7.0 Å, it is numbered as #3. For cutoff values above 8.5 Å, the aforementioned top 4 structures are classified within cluster #1; thus, the statistics are no longer reported here but in Supplementary Table 5C.

Supplementary Table 5 (*cont.*).

(D) $Ca_V\beta_4$–actin dimer models, cluster #5				
**i-l-RMSD cutoff (Å)**	**cluster size**	**i-l-RMSD (Å)**	**i-RMSD (Å)**	**l-RMSD (Å)**
5.0	8	4.4±1.4	0.4±0.2	4.3±1.4
5.5	10	4.4±2.4	0.5±0.5	4.4±2.5
6.0	11	4.9±2.7	0.7±0.7	4.9±2.8
6.5	11	4.9±2.7	0.7±0.7	4.9±2.8
7.0	11	4.9±2.7	0.7±0.7	4.9±2.8
7.5*	4	2.2±1.8	0.5±0.3	2.4±2.1
8.0	4	2.2±1.8	0.5±0.3	2.4±2.1
8.5	16	9.0±3.6	1.1±0.7	9.4±4.2
9.0	6	21.5±1.5	2.9±0.7	19.7±2.1
9.5	19	7.9±4.2	1.0±0.7	8.3±4.7
10.0	19	7.9±4.2	1.0±0.7	8.3±4.7

* For the top 4 models of the fifth cluster identified with a 7.5 Å i-l-RMSD cutoff, the i-l-RMSD, i-RMSD and l-RMSD values are the same as listed in the table above, as the cluster size contains only these four structures.

N.B. The cluster analyzed for each of the i-l-RMSD cutoff values listed above is the one containing the top 4 structures of the fifth cluster identified with a 7.5 Å i-l-RMSD cutoff (as shown in the Supplementary Table 5D header). For the cutoff range 5.0-7.0 Å and 8.0 Å, the corresponding cluster is #4, whereas for cutoff values 8.5-9.0 Å and 9.5-10.0 Å, it is numbered as #3 and #2, respectively.

Supplementary Table 6. Control protein-protein docking simulations without XL-MS-based distance restraints. The same protein structures as in the XL-MS guided docking presented in Supplementary Table 4A were used, but sampling of docking solutions was increased to 10,000 (rigid body docking) and 400 (simulated annealing and water refinement steps). The *ab initio* docking options listed below were employed in place of the XL-MS-based distance restraints to ensure the contact between the two proteins; several control simulations were run, with different combinations of such *ab initio* docking options. Regardless of the *ab initio* docking settings used, the resulting models were largely inconsistent with the experimentally identified interXLs; depending on the control simulation, only between 0 and 6 models (out of 400 generated in each docking) were consistent with the XL-MS data. The best scored *ab initio* docking models (top 1 and top 10, ranking based on the HADDOCK score) were also significantly different from the XL-MS guided (and experimentally validated) actin–$Ca_v\beta_2$ complex model, as shown by their I-RMSD values.

Control simulation #	***Ab initio* docking settings**[(a)]			**Docking comparison**[(b)]			**InterXL consistency**[(c)]	
	**Center of mass restraints**	**Random AIRs**	**Surface contact restraints**	**Top 1 scored model I-RMSD (Å)**	**Top 10 scored models I-RMSD (Å)**	**Closest model I-RMSD (Å) & ranking position**	**# Models consistent with interXLs**	**XL consistent models I-RMSD (Å)**
1	yes	yes	yes	78.9	69.8±9.5	14.0, #208[(d)]	4	91.7, 14.0<br>81.2, 87.1
2	yes	no	yes	66.6	72.6±6.5	19.9, #248	6	91.4, 83.7<br>83.7, 83.8<br>83.7, 45.6
3	yes	yes	no	64.4	77.0±19.6	26.0, #150	3	91.8, 83.9<br>81.2
4	no	yes	yes	79.8	70.5±10.8	31.7, #35	3	82.5, 79.9<br>83.1
5	no	no	yes	73.9	70.6±12.8	33.4, #344	2	95.2, 62.3
6	no	yes	no	55.3	67.0±26.5	18.5, #10	0	–

[(a)] The following *ab initio* docking options available in the HADDOCK webserver were used. (i) Center of mass restraints are defined between each molecule as an ambiguous distance restraint with centers averaging between all Cα atoms of each molecule. The upper distance limit is automatically defined as the sum of the “effective radius” of each molecule, which is in turn defined as half the average length of the three principal components. (ii) Random ambiguous interaction restraints (AIRs) are based on solvent accessible residues (>20% relative accessibility). During the rigid body docking step, such AIRs are defined from a 5 Å radius patch randomly selected from one protein to a 7.5 Å radius patch randomly selected on the other molecule and vice versa, whereas in the simulated annealing step the AIRs are automatically defined between all residues within 5 Å across the interface.(iii) Surface contact restraints are defined between each molecule as an ambiguous distance restraint with sum averaging between all Cα atoms of one protein and all Cα atoms of the other protein; the upper distance limit is set to 7 Å.

[(b)] The *ab initio* docking models turned out to be very heterogeneous, with clustering (using the default i-l-RMSD cutoff of 7.5 Å) resulting in either a single cluster containing only 1% of the 400 structures generated or a large number of clusters (between 17 and 28, depending on the control simulation) covering only a subset of docking models (between 9 and 54%). Thus, for the analyses presented here we chose to consider all 400 *ab initio* models without clustering. As the docking between the actin dimer and $Ca_v\beta_2$ is a multibody docking simulation of a ternary complex, some of the generated models showed a disrupted actin dimer interface. Hence, we first filtered the generated 400 actin–$Ca_v\beta_2$ complex models based on the actin backbone RMSD with respect to the initial structure of the actin dimer alone; only those models with RMSD below 3.5 Å were retained for further analysis. We then compared the two docking protocols (XL-MS-guided and *ab initio*) using two different approaches. The first comparison was performed by selecting the best scored *ab initio* docking models, either the first (top 1) or the first ten (top 10) structures ranked by HADDOCK score, and calculating their l-RMSD (see Methods), using as reference the best scored XL-MS-guided docking model. The second comparison consisted in identifying the *ab initio* docking model with the lowest l-RMSD with respect to the best scored XL-MS-guided docking model and reporting its l-RMSD value together with the HADDOCK score-based ranking position among the 400 models generated in the corresponding control simulation.

[(c)] Models were considered to be consistent with XL data if the Euclidean distances of all 11 identified inter-crosslinks (see Supplementary Table 3A) were lower or equal to 35.0 Å. The l-RMSD was calculated as explained in the Methods, using as reference the best scored actin–$Ca_v\beta_2$ complex model obtained in the XL-MS-guided docking simulation.

[(d)] It is worth mentioning that one of the models of control simulation #1 was consistent with the XL-MS data and, with respect to the best XL-MS-guided docking model, displayed the lowest l-RMSD value (14.0 Å) across all six control simulations. However, this *ab initio* model was ranked 208 (out of 400 models in control simulation #1) and thus would not have been selected based on the HADDOCK score alone. Moreover, the superposition of this *ab initio* model with the XL-MS guided actin–$Ca_v\beta_2$ complex model (see Supplementary Figure 5) showed that the protein-protein interface of the two complexes is different, as evidenced by the i-RMSD value of 7.9 Å.

Supplementary Table 7. Intermolecular contacts involving the eight Ca$_V$β hotspot residues at the protein-protein interface with actin. The top 4 models of the first cluster of the Ca$_V$β-actin dimer docking simulations were analyzed with the PRODIGY webserver. Actin residues in monomer *n+2* are indicated with a prime (') symbol. Contacts observed/absent in the corresponding model are indicated with a tick/cross, respectively.

(A) Ca$_V$β$_2$ hotspot–actin contacts					
**Ca$_V$β2 hotspot**	**Actin interacting residue**	**Top1 structure**	**Top 2 structure**	**Top 3 structure**	**Top 4 structure**
**K90**	T7	×	×	×	✓
	D26	✓	✓	✓	✓
	D27	✓	×	✓	×
	A28	✓	×	✓	×
	R30	×	✓	✓	✓
**R128**	Y145	✓	×	✓	×
	I347	✓	×	✓	×
	L348	✓	×	✓	×
	A349	×	✓	×	✓
	S350	✓	✓	✓	✓
	L351	✓	✓	✓	✓
	S352	×	✓	✓	✓
	Q355	×	✓	×	✓
**K347**	G148’	✓	✓	✓	×
	R149’	✓	✓	✓	×
	T150’	✓	✓	✓	×
	T151’	✓	✓	✓	×
	Y168’	✓	✓	✓	×
	E169’	✓	✓	✓	×
	K293’	×	×	✓	×
	D294’	✓	✓	✓	×
	N298’	✓	×	✓	×
**Q350**	L144’	×	✓	×	✓
	Y145’	×	✓	×	×
	S147’	×	✓	×	×
	G148’	✓	✓	✓	✓
	R149’	✓	✓	✓	✓
	T150’	✓	✓	✓	✓
	T151’	×	×	✓	×
**K354**	L144’	×	✓	×	×
	Y145’	✓	✓	✓	✓
	A146’	✓	✓	✓	✓
	S147’	✓	✓	✓	✓
	G148’	✓	✓	✓	✓
	R149’	×	✓	×	×
	I343’	×	✓	×	×
	I347’	✓	✓	✓	✓

– Continued in the next page –

(A) $Ca_V\beta_2$ hotspot–actin contacts (*cont.*)					
**$Ca_V\beta 2$ hotspot**	**Actin interacting residue**	**Top1 structure**	**Top 2 structure**	**Top 3 structure**	**Top 4 structure**
**K358**	G25’	√	√	√	√
	D26’	√	√	√	√
	D27’	√	√	√	√
	A28’	×	×	×	√
	S346’	√	×	×	×
	I347’	√	√	√	×
	S350’		√	×	×
**L364**	Y145’	√	×	×	×
	L351’	√	×	×	×
	S352’	√	×	×	×
**N365**	V45	√	×	√	×
	G48	√	×	√	×
	M49	√	×	×	×
	Y145’	×	×	√	×
	L351’	√	√	√	√
	S352’	√	√	×	×
	T353’	√	√	√	√

Supplementary Table 7 (*cont.*).

(B) Ca$_V\beta_4$ hotspot–actin contacts					
**Ca$_V\beta_4$ hotspot**	**Actin interacting residue**	**Top1 structure**	**Top 2 structure**	**Top 3 structure**	**Top 4 structure**
**K122**	G25	×	×	×	✓
	D26	✓	×	×	✓
	D27	✓	×	×	✓
	A28	✓	×	×	✓
	R30	×	×	✓	×
**R160**	T8	×	✓	✓	×
	A24	×	✓	✓	×
	G25	×	✓	✓	×
	D26	×	✓	✓	×
	S350	✓	×	×	✓
	L351	✓	×	×	✓
	S352	✓	×	×	✓
**K339**	P245	×	×	×	✓
	R149’	✓	✓	✓	
	K293’	✓	×	×	✓
	D294’	✓	×	×	✓
	L295’	✓	×	×	
	Y296’	×	×	×	✓
	A297’	✓	×	×	✓
	N298’	✓	×	×	
	M327’	×	×	×	✓
	K328’	×	×	×	✓
	K330’	×	✓	×	×
	I331’	×	✓	✓	×
	I332’	×	✓	✓	×
	A333’	×	✓	✓	×
**Q342**	A146’	×	✓	✓	×
	S147’	✓	✓	✓	×
	G148’	✓	✓	✓	×
	R149’	✓	✓	✓	×
	T150’	✓	×	×	×
	D294’	×	×	×	✓
	A297’	×	×	×	✓
	N298’	×	×	×	✓
	I332’	×	✓	✓	×
	P334’	×	✓	✓	×

– Continued in the next page –

(B) Ca$_V$β$_4$ hotspot–actin contacts (*cont.*)					
**Ca$_V$β$_4$ hotspot**	**Actin interacting residue**	**Top1 structure**	**Top 2 structure**	**Top 3 structure**	**Top 4 structure**
**K346**	D27’	×	✓	✓	×
	A146’	✓	×	×	×
	S147’	✓	×	×	×
	G148’	✓	×	×	×
	R149’	×	×	×	✓
	A297’	×	×	×	✓
	N298’	×	×	×	✓
	I332’	×	×	×	✓
	P335’	×	✓	✓	×
	E336’	×	✓	✓	×
	I342’	×	×	✓	×
**K350**	G25’	×	✓	✓	×
	D26’	×	✓	✓	×
	D27’	×	✓	✓	×
	A28’	×	✓	✓	×
	R30’	×	✓	✓	×
	A146’	×	×	×	✓
	S147’	×	×	×	✓
	I343’	×	×	×	✓
	I347’	×	×	×	✓
**L356**	Y145’	×	✓	✓	×
	A146’	×	✓	✓	×
	S147’	×	✓	✓	×
	G148’	✓	✓	✓	✓
	R149'	✓	×	✓	✓
	I347’	×	✓	×	×
**N357**	V45	✓	✓	✓	×
	G48	✓	×	×	×
	M49	✓	×	×	×
	L144’	×	✓	×	×
	Y145’	×	✓	✓	×
	A146’	×	✓	×	×
	S147’	×	✓	×	×
	G148’	×	✓	✓	✓
	R149’	×	✓	✓	✓
	T150’	✓	✓	✓	✓
	T151’	×	×	×	✓
	E169’	×	×	×	✓
	I347’	×	✓	×	×

Supplementary Table 8. Non-synonymous SNPs of human $Ca_V\beta_2$ and $Ca_V\beta_4$ at amino acid positions analogous to the eight $Ca_V\beta$ hotspot residues at the protein-protein interface with actin.
(**A**) Human $Ca_V\beta_2$ (www.uniprot.org/uniprotkb/Q08289/variant-viewer). (**B**) Human $Ca_V\beta_4$ (www.uniprot.org/uniprotkb/O00305/variant-viewer). Data retrieved on November 2024 from the UniProt database.

(A) Non-synonymous SNPs for $Ca_V\beta_2$ actin hotspots					
**rat $Ca_V\beta_2$ (Q8VGC3-2)**	**human $Ca_V\beta_2$ (Q082289-1)**	**Variant ID**	**Change**	**Description & Clinical Significance**	**Provenance**
K90	K145	rs1554831385	K>*		Ensembl
R128	R183	TCGA novel	R>S	Variant assessed as somatic; MODERATE impact (NCI-TCGA)	NCI-TCGA
		rs2050158937	R>G		TOPmed
K347	K402	rs1554840899	K>*		Ensembl
Q350	Q405	rs1554841459	Q>*		Ensembl
		rs1206230612	Q>H		TOPmed gnomAD
		rs1281052136	Q>R		TOPmed
K354	K409	rs1554841468	K>*		Ensembl
		TCGA novel	K>Q	Variant assessed as somatic; MODERATE impact (NCI-TCGA)	NCI-TCGA
		rs2053546700	K>R		TOPmed
K358	K413	rs1554841479	K>*		Ensembl
L364	L419	rs1202561047	L>P		gnomAD
N365	N420	COSV56627386	N>S	Variant assessed as somatic; MODERATE impact (NCI-TCGA)	NCI-TCGA Cosmic cosmic curated

Supplementary Table 8 (*cont.*).

(B) Non-synonymous SNPs for $Ca_V\beta_4$ actin hotspots					
**rat CaVβ4 (D4A055-2)**	**human CaVβ4 (O00305-1)**	**Variant ID**	**Change**	**Effect**	**Provenance**
K122	K123	rs761287173	K>E		ExAC<br>gnomAD
		rs753508642	K>T		ExAC<br>gnomAD
R160	R161	rs1199094751	R>Q		gnomAD
		COSV52393347<br>rs1257214495	R>W	Variant assessed as somatic; MODERATE impact (NCI-TCGA)	NCI-TCGA<br>Cosmic<br>cosmic curated<br>TOPmed<br>dbSNP<br>gnomAD
K339	K340	rs1553746911	K>*		Ensembl
Q342	Q343	rs1328517680	Q>*		TOPmed<br>gnomAD
		CA348789818<br>RCV000634979<br>rs1328517680	Q>E	Idiopathic generalized epilepsy (ClinVar)<br>Variant of uncertain significance (Ensembl, ClinVar)	ClinGen<br>ClinVar<br>TOPMed<br>dbSNP<br>gnomAD
K346	K347	rs1169198515	K>*		gnomAD
		rs1169198515	K>E		gnomAD
K350	K351	rs1553746086	K>*		Ensembl
L356	L357	rs1553746054	L>*		Ensembl
N357	N358	rs746420663	N>S		ExAC<br>gnomAD

Supplementary Table 9

REAGENT or RESOURCE	SOURCE	IDENTIFIER
Antibodies		
anti-CACNB2	Novus Biologicals	NBP186680
anti-GADPH	Sigma-Aldrich	G9545
anti-CACNB4	Novus Biologicals	NBP212908
goat anti-rabbit IgG HRP-conjugated	Thermo Fisher Scientific	31463
Bacterial and cell lines		
BL21 (DE3) competent *E.coli* cells (recombinant protein production)	Thermo Scientific	Cat #EC0114
TOP10F competent *E.coli* cells (molecular biology)	Invitrogen	Cat#C303006
HEK293	Sigma-Aldrich	Cat #12022001
Chemicals		
Cesium methanesulfonate	Sigma	Cat#C1426
EGTA	Sigma	Cat#E3889
Cesium chloride	Sigma	Cat#289329
Magnesium chloride·6H2O	Serva	Cat#39771.01
HEPES	PanReac	Cat#A1069
Cesium hydroxide solution	Sigma	Cat#232041
Barium chloride dihydrate	Sigma	Cat#B0750
Tetraethylammonium hydroxide solution	Sigma	Cat#86633
CellMask™ Plasma Membrane Stain Green	Thermo Fisher Scientific	Cat#C37608
Opti-MEM™	Thermo Fisher Scientific	Cat#11058021
DMEM, high glucose, GlutaMAX™ Supplement	Thermo Fisher Scientific	Cat#61965026
Penicillin-Streptomycin (5,000 U/mL)	Thermo Fisher Scientific	Cat#15070063
Fetal Bovine Serum	Thermo Fisher Scientific	Cat#10270106
Dimethylsulfoxide (DMSO) anhydrous	Invitrogen	Cat#D12345
DSBU (Disuccinimidyl Dibutyric Urea)	Thermo Fisher Scientific	Cat#A35459
DSSO (Disuccinimidyl Sulfoxide)	Thermo Fisher Scientific	Cat#A33545
cOmplete™ EDTA-free Protease Inhibitor Cocktail	Roche	Cat#05056489001
Rabbit skeletal muscle actin	Cytoskeleton	Cat#AKL99

Trypsin	SERVA	Cat#37286.01
