# Supplementary material for: Mapping the interaction surface between CaVβ and actin and its role in calcium channel clearance
Source: Nat Commun. 2025 May 10;16:4352. doi: 10.1038/s41467-025-59548-x (PMC12065904; doi:10.1038/s41467-025-59548-x)
Supplement: Supplementary file 2 — Reporting Summary [file 41467_2025_59548_MOESM2_ESM.pdf]

nature portfolio

Corresponding author(s): Patricia Hidalgo
Beatrix Santiago-Schübel
Mercedes Alfonso-Prieto

Last updated by author(s): Apr 9, 2025

Reporting Summary

Nature Portfolio wishes to improve the reproducibility of the work that we publish. This form provides structure for consistency and transparency in reporting. For further information on Nature Portfolio policies, see our Editorial Policies and the Editorial Policy Checklist.

Statistics

For all statistical analyses, confirm that the following items are present in the figure legend, table legend, main text, or Methods section.

n/a	Confirmed	
☐	☒	The exact sample size (n) for each experimental group/condition, given as a discrete number and unit of measurement
☐	☒	A statement on whether measurements were taken from distinct samples or whether the same sample was measured repeatedly
☐	☒	The statistical test(s) used AND whether they are one- or two-sided<br>*Only common tests should be described solely by name; describe more complex techniques in the Methods section.*
☒	☐	A description of all covariates tested
☐	☒	A description of any assumptions or corrections, such as tests of normality and adjustment for multiple comparisons
☐	☒	A full description of the statistical parameters including central tendency (e.g. means) or other basic estimates (e.g. regression coefficient) AND variation (e.g. standard deviation) or associated estimates of uncertainty (e.g. confidence intervals)
☐	☒	For null hypothesis testing, the test statistic (e.g. F, t, r) with confidence intervals, effect sizes, degrees of freedom and P value noted<br>*Give P values as exact values whenever suitable.*
☒	☐	For Bayesian analysis, information on the choice of priors and Markov chain Monte Carlo settings
☒	☐	For hierarchical and complex designs, identification of the appropriate level for tests and full reporting of outcomes
☒	☐	Estimates of effect sizes (e.g. Cohen's d, Pearson's r), indicating how they were calculated

Our web collection on statistics for biologists contains articles on many of the points above.

Software and code

Policy information about availability of computer code

Data collection	PatchMaster v2x90.5<br>Leica Application Suite, LAS AF Version 2.7.3.9723<br>HADDOCK 2.4
Data analysis	msconvert GUI - ProteoWizard Toolkit version 3<br>MaxLynx version 2.2.0.0<br>MetaMorpheus version 1.0.2<br>MeroX version 2.0<br>CPORT version 0.2.0<br>DisVis version 2.3.0<br>NACCESS version 2.1.1.<br>VMD version 1.9.3<br>Jupyter Notebook version 6.5.4<br>Anchor version 2012<br>BeAtMuSiC version 2013<br>BUDE version 1.0<br>Mutabind2 version 2<br>Robetta version 2011

SAAMBE_3D version 2020
Python 3.8
Python libraries numpy version 1.24, matplotlib version 3.7 and pandas 2.1
Origin Pro 2022
Microsoft Excel 2019
ImageJ 1.44p
APBS version 3.4.1
PRODIGY version 2.2.2

For manuscripts utilizing custom algorithms or software that are central to the research but not yet described in published literature, software must be made available to editors and reviewers. We strongly encourage code deposition in a community repository (e.g. GitHub). See the Nature Portfolio guidelines for submitting code & software for further information.

Data

Policy information about availability of data

All manuscripts must include a data availability statement. This statement should provide the following information, where applicable:

- Accession codes, unique identifiers, or web links for publicly available datasets
- A description of any restrictions on data availability
- For clinical datasets or third party data, please ensure that the statement adheres to our policy

Raw mass spectrometry and search data have been deposited to the ProteomeXchange Consortium (http://proteomecentral.proteomexchange.org) via the PRIDE partner repository134 with the dataset identifiers PXD053456 (DSBU) and PXD053481 (DSSO) respectively.
Data required to reproduce the docking results and resulting CaVβ-F/actin complex models, along with the computational source data, are available at Zenodo (https://doi.org/10.5281/zenodo.8276447).
Electrophysiology and in vitro assays data https://jugit.fz-juelich.de/HidalgoPatricia/beta-actin_interaction_surface

Research involving human participants, their data, or biological material

Policy information about studies with human participants or human data. See also policy information about sex, gender (identity/presentation), and sexual orientation and race, ethnicity and racism.

Reporting on sex and gender	n/a
Reporting on race, ethnicity, or other socially relevant groupings	n/a
Population characteristics	n/a
Recruitment	n/a
Ethics oversight	n/a

Note that full information on the approval of the study protocol must also be provided in the manuscript.

Field-specific reporting

Please select the one below that is the best fit for your research. If you are not sure, read the appropriate sections before making your selection.

[x] Life sciences [ ] Behavioural & social sciences [ ] Ecological, evolutionary & environmental sciences

For a reference copy of the document with all sections, see nature.com/documents/nr-reporting-summary-flat.pdf

Life sciences study design

All studies must disclose on these points even when the disclosure is negative.

Sample size	The sample size for the docking models of either Cavβ/actin complex was 200; however, the initial rigid body docking step generated 1,000 initial models, out of which the top 200 best scored models are selected for subsequent semi-flexible simulated annealing and fully flexible refinement to obtain the final 200 models. We chose a sample size of n ≥ 10 based on previous publications and sample sizes common in electrophysiology studies using transiently transfected cells. The results of the biochemical assays (Fig. 3D; 4C,F; Fig. 5B,D,F; Fig. 6C and Fig. 8B) were obtained from three independent experiments following the standard practice. Confocal imaging was produced from two separate cell transfections.
Data exclusions	The XL-MS-derived distance restraints identified as false positives by DisVis filtering were excluded from the subsequent XL-MS-guided protein-protein docking. Recordings were excluded if they had a seal resistance below 2 GOhm, compensated series resistance above 3 MOhm, or were incomplete due to seal loss or membrane rupture.

Replication	Cross linking reactions were performed for DSSO and DSBU in 5 and 4 replicates, respectively.
Randomization	No randomization was applied, except for the HADDOCK protein-protein docking protocol, starting orientations the Cavβ and actin are randomized by translation and rotation before rigid body docking. Moreover, 50% of the ambiguous interaction restraints are randomly excluded in each docking trial
Blinding	The investigator was blinded for the electrophysiological recordings with respect to the combination of cotransfected vectors encoding the CaV calcium channel subunits. Blinding was not relevant for the biochemical assays as the use of standarized protocols and instrumental readout reduces the potential for experimental bias.

Reporting for specific materials, systems and methods

We require information from authors about some types of materials, experimental systems and methods used in many studies. Here, indicate whether each material, system or method listed is relevant to your study. If you are not sure if a list item applies to your research, read the appropriate section before selecting a response.

Materials & experimental systems

n/a	Involved in the study
☐	☒ Antibodies
☐	☒ Eukaryotic cell lines
☒	☐ Palaeontology and archaeology
☒	☐ Animals and other organisms
☒	☐ Clinical data
☒	☐ Dual use research of concern
☒	☐ Plants

Methods

n/a	Involved in the study
☒	☐ ChIP-seq
☒	☐ Flow cytometry
☒	☐ MRI-based neuroimaging

Antibodies

Antibodies used	anti-CACNB2 Novus Biologicals NBP186680<br>anti-CACNB4 Novus Biologicals NBP212908<br>anti-GADPH Sigma G9545<br>goat anti-rabbit IgG HRP-conjugated Pierce 31463
Validation	All antibodies used in this study were comercially available and validated by the manufacturers

Eukaryotic cell lines

Policy information about cell lines and Sex and Gender in Research

Cell line source(s)	HEK293, Sigma-Aldrich, Cat #12022001.<br>BL21 (DE3) competent cells, Thermo Scientific, Cat #EC0114.
Authentication	HEK293 and BL21 (DE3) cells were obtained directly from the providers and were not authenticated by us.
Mycoplasma contamination	HEK293 cells were tested negative for mycoplasma contamination.
Commonly misidentified lines<br>(See ICLAC register)	No commonly misidentified lines were used.

Plants

Seed stocks	n/a
Novel plant genotypes	n/a
Authentication	n/a
